# Amphiphilic “Like-A-Brush” Oligonucleotide Conjugates with Three Dodecyl Chains: Self-Assembly Features of Novel Scaffold Compounds for Nucleic Acids Delivery

**DOI:** 10.3390/nano10101948

**Published:** 2020-09-29

**Authors:** Anna S. Pavlova, Ilya S. Dovydenko, Maxim S. Kupryushkin, Alina E. Grigor’eva, Inna A. Pyshnaya, Dmitrii V. Pyshnyi

**Affiliations:** Institute of Chemical Biology and Fundamental Medicine SB RAS, 630090 Novosibirsk, Russia; pavlova@niboch.nsc.ru (A.S.P.); dovydenko_il@niboch.nsc.ru (I.S.D.); kuprummax@niboch.nsc.ru (M.S.K.); grigoryeva@niboch.nsc.ru (A.E.G.); pyshnaya@niboch.nsc.ru (I.A.P.)

**Keywords:** lipid conjugates, amphiphilic oligonucleotides, self-assembly, phosphoryl guanidines, nucleic acid delivery

## Abstract

The conjugation of lipophilic groups to oligonucleotides is a promising approach for improving nucleic acid-based therapeutics’ intracellular delivery. Lipid oligonucleotide conjugates can self-aggregate in aqueous solution, which gains much attention due to the formation of micellar particles suitable for cell endocytosis. Here, we describe self-association features of novel “like-a-brush” oligonucleotide conjugates bearing three dodecyl chains. The self-assembly of the conjugates into 30–170 nm micellar particles with a high tendency to aggregate was shown using dynamic light scattering (DLS), atomic force (AFM), and transmission electron (TEM) microscopies. Fluorescently labeled conjugates demonstrated significant quenching of fluorescence intensity (up to 90%) under micelle formation conditions. The conjugates possess increased binding affinity to serum albumin as compared with free oligonucleotides. The dodecyl oligonucleotide conjugate and its duplex efficiently internalized and accumulated into HepG2 cells’ cytoplasm without any transfection agent. It was shown that the addition of serum albumin or fetal bovine serum to the medium decreased oligonucleotide uptake efficacy (by 22.5–36%) but did not completely inhibit cell penetration. The obtained results allow considering dodecyl-containing oligonucleotides as scaffold compounds for engineering nucleic acid delivery vehicles.

## 1. Introduction

Synthetic oligonucleotide conjugates with lipophilic groups are in the focus of considerable attention as nucleic acid (NA)-based biological tools in a wide range of fields of biotechnology and biomedicine [1,2]. Lipid-oligonucleotide conjugates (LOCs) are particularly interesting as delivery vehicles, which bring therapeutic oligonucleotides (e.g., antisense oligomers or siRNA) to their intracellular targets [3,4,5,6]. Negatively charged native oligonucleotides show poor cell penetration [5,6]. Their conjugation with lipophilic moieties can improve cellular uptake by providing an additional anchor for membrane binding [7,8,9,10,11].

Due to their amphipathic nature, oligonucleotide conjugates with lipophilic groups can self-assemble in aqueous solutions and form micelles [10,12,13,14,15,16,17,18] and their aggregates [10,11,14], vesicular assemblies [19,20], and more complex self-aggregation structures [2,11,21]. Generally, these structures are nearly spherical in shape [10,15,16,20,22,23]. The size and the shape of self-assembling structures may greatly depend on experimental conditions: ionic strength, pH, and temperature; the type of lipophilic group; the length and nucleotide sequence of an oligomer in the LOC [2,17].

Cargo systems can be based as well on the attachment of lipophilic groups directly to a specific antisense oligonucleotide [10,24,25], to one of the siRNA strands [11,21,26,27] or more complex delivery formulations [13,23,28,29]. Furthermore, LOCs suit for the construction of oligonucleotide delivery system based on the liposomal-type spherical nucleic acids (SNA) [30,31,32]. The hydrophobic micellar core of LOC particles also serves as a carrier for extremely poor soluble pharmaceuticals [14,23,29] and fluorescent dyes [13,19].

Substantial characteristics of lipid-oligonucleotide conjugates include good biocompatibility and low toxicity [10,11,33]. LOCs improve pharmacokinetics and biodistribution of antisense oligomers (ASO) compared with native unconjugated oligonucleotides [4]. This advance is generally attributed to enhanced LOC binding to serum proteins. The association of the LOCs with one or more serum proteins (albumin, etc.) is also believed to participate in their cellular uptake mechanisms [4]. Albumin is the major blood protein that binds and transports numerous endogenous and exogenous substances, including fatty acids and other poor water-soluble compounds [34]. Lipid-oligonucleotide conjugates can easily bind as well to hydrophobic sites of the albumin [5,22]. It appears to affect their circulation half-life and bioavailability, e.g., for intravenous administration of LOC-based pharmaceuticals. Modifications of the oligonucleotide backbone (phosphorothioate (PS), morpholino, peptide nucleic acids, 2′-*O*-methyl, 2′-fluoro, and others) combined with lipophilic group enhance nuclease resistance of oligonucleotides, thus increasing their in vivo circulation lifetime [4,5,6].

So far, cholesterol is one of the most investigated lipophilic groups conjugated with oligonucleotides to improve cellular uptake [4,5,8]. Other promising modifications of this kind are fatty acids and lipid chains [6]. For example, Imetelstat (GRN163L), a palmitoyl-tethered thio-phosphoramidate oligomer, provides telomerase inhibition in the treatment of myeloproliferative disorders or neoplasms [6,24]. An interesting type of conjugates is represented by LOCs with “like-a-brush” lipid moieties attached to the oligonucleotide backbone [10,14,20,35]. Conjugation in such a manner gives the rise of hydrophobicity of the LOC lipophilic segment, in contrast to the consecutive linear combination. Earlier, we designed a non-nucleoside monomer to incorporate dodecyl groups into the oligonucleotides and synthesized dodecyl-containing conjugates with hydrophobicity comparable to that of corresponding cholesterol derivatives [36]. It was recently demonstrated that three dodecyl oligonucleotide conjugates (DOCs) are efficient and non-toxic transport molecules for ASO delivery into the A549 and HEK293 cells, which provide the transfection efficacy comparable to that of Lipofectamine 2000 [33].

In the present work, we report on self-assembly features of amphiphilic “like-a-brush” oligonucleotide conjugates functionalized at 5′ and 3′-ends with three dodecyl groups [36]. These DOCs were studied for their abilities to form micellar structures and to penetrate the HepG2 tumor cells. In vitro investigation of self-assembling features of DOCs by dynamic light scattering (DLS), electrophoretic mobility shift assay (EMSA), fluorescence spectroscopy, transmission electron (TEM) and atomic force (AFM) microscopies, flow cytometry, and confocal microscopy substantially extended current knowledge on micelle-like structures of “like-a-brush” lipophilic oligonucleotide conjugates. The obtained results establish the potential of DOCs as nucleic acid delivery tools.

## 2. Materials and Methods

### 2.1. General Remarks

Buffers composition: TA—50 mM Tris-Acetate, pH 7.5; TAM—50 mM Tris-Acetate, pH 7.5, 15 mM MgCl_2_; TAN—50 Tris-Acetate, pH 7.5, 100 mM NaCl. All buffers were filtered through 0.22 µm Millipore Syringe Filter units (Merck, KGaA, Darmstadt, Germany, and/or its affiliates). Oligonucleotides and DOCs were incubated in 1.5 mL DNA LoBind tubes (Eppendorf AG, Hamburg, Germany).

### 2.2. Materials

Nile Red (9-diethylamino-5H-benzo[alpha]phenoxazine-5-one (C_20_H_18_N_2_O_2_), Merck, KGaA, Darmstadt, Germany, and/or its affiliates), bovine serum albumin (BSA) (Sigma-Aldrich Chemie Gmbh, Munich, Germany), Stains-All (Acros Organics, Fair Lawn, NJ, USA). Unless otherwise stated, all commercial reagents and solvents were used without additional purification. All chemicals used in this work were molecular biology grade or higher. Water was 18 MΩ grade (purified by a Simplicity 185 water system (Millipore, Burlington, MA, USA).

### 2.3. Oligonucleotides and Conjugates Synthesis

Standard phosphoramidite solid-phase synthesis of all modified/unmodified and conjugated/unconjugated oligonucleotides was carried out on the ASM-800 DNA/RNA synthesizer (Biosset, Novosibirsk, Russia). Oligonucleotides were synthesized at 0.2 µmol scale, using standard commercial 2-cyanoethyl deoxynucleoside phosphoramidites and CPG solid supports (Glen Research, Sterling, VA, USA). The phosphoramidite for introducing non-nucleosidic dodecyl-containing units was obtained as described [36] and used as a 0.1 M solution in anhydrous acetonitrile with the extension of the coupling time from 1 to 10 min. Oligonucleotides with internucleoside uncharged phosphoryl 1,3-dimethylimidazolidine-2-imino groups (phosphoryl guanidines, PG) were synthetized by NooGen LLC as described earlier [37,38,39]. 6-carboxyfluorescein (FAM) labeling of the oligonucleotides was performed using commercially available 5′- and 3′-modifiers (2-Dimethoxytrityloxymethyl-6-(3′,6′-dipivaloylfluorescein-6-yl-carboxamido)-hexyl-1-*O*-[(2-cyanoethyl)-(*N*,*N*-diisopropyl)]-phosphoramidite from Glen Research, Sterling, VA, USA and 3′-FAM-CPG (Figure S1) from Primetech ALC, Minsk, Belarus) according to the manufacturer’s protocols. All oligomers after cleavage and deblocking from CPG were purified by RP-HPLC and their structures (for examples see Figures S3–S5) were confirmed by MALDI TOF or ESI mass spectrometry (for additional information see Supp. Inf., Section S1, Table S1, Figure S6).

### 2.4. Critical Aggregation Concentration (CAC) Determination by Nile Red Encapsulation Assay

Formation of the DOC micelles was characterized as described in the literature [23], using Nile Red as a fluorescent probe. A 10 mM stock solution of the Nile Red in ethanol was used for all experiments. Briefly, 0.7 µM, 1 µM, 3 µM, 10 µM, 30 µM, and 50 µM of the DOC or control oligonucleotide were incubated in eppendorfs with 100 µM Nile Red in TA or TAM buffer at 25 °C for 3 h. After incubation, time samples were transferred in TPP^®^ tissue culture plates (Sigma-Aldrich Chemie GmbH, Buchs, Switzerland). The fluorescence intensity spectra of the Nile Red were obtained at room temperature using a CLARIOStar^®^ Microplate reader (BMG LABTECH GmbH, Ortenberg, Germany). Fluorescent measurements were taken at the excitation wavelength of 550 nm and the emission was monitored from 570 to 740 nm. The oligonucleotide without dodecyl chains was used as a control. The critical aggregation concentration (CAC) could be calculated by tracking the fluorescence intensity of Nile Red as a function of the sample concentration. CAC values were calculated from the plot of the emission intensity at 645 nm (in TA buffer) or 630 nm (in TAM) versus the log of concentrations (M) of dodecyl oligonucleotide conjugates. The CAC was obtained from the intersection of two straight tangents to these regions’ lines.

### 2.5. Characterization of Assembled DOCs Micellar Structures by DLS

The size distributions of micellar particles of pre-assembled DOCs were determined by dynamic light scattering technique using a Zetasizer Nano-ZS (Malvern Panalytical Ltd., Malvern, UK) at 25 °C. The DOCs (5 µM) were prepared in TAM buffer and after 3 h of incubation measurements of the size were conducted.

### 2.6. Characterization of Assembled DOCs Micellar Structures by AFM

Atomic force microscopy was performed using a MultiMode 8™ scanning probe microscope (Bruker, Santa Barbara, CA, USA) connected to a NanoScope^®^ V controller (Veeco, Plainview, NY, USA). The images were obtained using tapping mode in air with NSG10_DLC cantilevers (typical curvature radius 1 nm, resonant frequency 255 kHz, force constant 11.5 N/m) from NT-MDT Spectrum Instruments (Zelenograd, Moscow, Russia). Oligonucleotides containing dodecyl groups were diluted to 1.5 µM in TAM buffer. The reactions were equilibrated for 3 h at 25 °C before 6 µL of this solution was deposited onto a freshly cleaved mica surface (7 × 7 mm, NT-MDT Spectrum Instruments, Zelenograd, Moscow, Russia) and allowed to adsorb for 5 min. The surface was then washed thrice with 200 µL of 18 MΩ grade water and dried by strong argon flow. Samples were dried for 10 min prior to imaging.

### 2.7. Characterization of Assembled DOCs Micellar Structures by TEM

Dodecyl oligonucleotide conjugates were diluted to 5 µM in TAM buffer. The reactions were equilibrated for 3 h at 25 °C before a drop of this sample was adsorbed for 1 min on the copper grid covered with formvar film which was stabilized using carbon evaporation. Then excess of liquid was removed with filter paper, and a grid was placed for 5–10 s on a drop of 1% uranyl acetate, excess liquid was collected with filter paper. All grids were examined using a transmission electron microscope JEM-1400 (JEOL, Tokyo, Japan), and images were obtained using a Veleta (EM SIS, Münster, Germany) digital camera. All measurements were made using program package iTEM (EM SIS, Münster, Germany).

### 2.8. EMSA and BSA Binding Experiments

Electrophoretic mobility shift assay was carried out using Thermo Scientific^TM^ Owl^TM^ Dual-Gel Vertical Electrophoresis System (P8DS-2, Owl, Thermo Fisher Scientific Inc., Waltham, MA, USA) at 25, 35, or 37 °C, 7–8 W for 3–4 h. Briefly, control oligonucleotides and DOCs were diluted to the required concentration in 20 µl of corresponding buffer solution (TA, TAM, or TAN) and incubated for 2 h prior to loading onto the native PAAG. In BSA binding experiments, the protein was added to the oligonucleotides after 2 h of incubation and then the probes were additionally incubated for 1 h prior to loading onto the gel. After electrophoretic separation, the DNA and BSA bands were visualized by Stains-All staining and in the case of FAM-labeled oligonucleotides by scanning and recording the image using VersaDoc^TM^ MP 4000 Molecular Imager^®^ System (Bio-Rad, Hercules, CA, USA) after excitation at 488 nm. For Stains-All staining (0.05% (w/v) Stains-All in 50% (v/v) formamide), the gel after the run was stained in the dark chamber for 10 min. Destaining was accomplished by removing the gel from the staining solution and exposing it to the light until sufficient destaining had occurred. The gel was then immediately scanned using an Epson Perfection 4990 Photo scanner (Epson, Los Alamitos, CA, USA).

### 2.9. Fluorescence Quenching Experiments

1.5 µM FAM-labeled oligonucleotides and DOCs were prepared in 18 MΩ grade water, TA, or TAM buffer solutions. The samples were equilibrated for 3 h at 25 °C before transferring into the flat bottom 96-well microplates (TPP^®^, Sigma-Aldrich Chemie GmbH, Buchs, Switzerland) and measured through reading fluorescence intensity spectra on a CLARIOstar^®^ plate reader (BMG LABTECH GmbH, Ortenberg, Germany) in top-read mode, with excitation at 488 nm (16 nm bandpass) and emission scanning from 503 nm to 619 nm (10 nm bandpass). Wells for background subtraction contained all components except FAM-labeled nucleic acids. Reactions were set up in triplicate in each experiment. To check the fluorescence quenching, the values of the fluorescence intensities at 520 nm, representative of fluorescence emission maximums for this dye, were compared for each of the solution conditions. Quenching efficiency (QE) was defined as QE = (F_TAM_ × 100)/F_TA_, where F_TA_ is the fluorescence intensity value of the oligomer in TA buffer, and F_TAM_—in TAM buffer, respectively.

### 2.10. Cell Culture

For all experiments, we used the hepatocellular carcinoma (HepG2) cell line obtained from Russian Cell Culture Collection, Institute of Cytology of the Russian Academy of Science (St. Petersburg, Russia). Cells were cultivated at 37 °C and 5% CO_2_ in DMEM medium with GlutaMAX^TM^ containing 4.5 g/L glucose, supplemented with 10% fetal bovine serum (FBS), 100 U/mL penicillin, 100 µg/mL streptomycin (all from Gibco^TM^ by Life Technologies^®^ Corporation, Paisley, UK).

### 2.11. Cells Transfection

One day before the carrier-free transfection procedure, cells were seeded in 24-well plates at a density of 1.2 × 10^5^ cells/well. Then cells were washed with PBS and treated for 4 h with a medium containing fluorescently labeled dodecyl oligonucleotide conjugate FAM-D-17^PG^ or duplex D-17^PG^/FAM-17′ (250 µL/well). We used six versions of the medium composition for cell transfection procedure: (a,d) fresh DMEM (245 µL) and FAM-D-17^PG^ or D-17^PG^/FAM-17′ (5 µL, 250 µM); (b,e) fresh DMEM with 10% FBS (245 µL) and FAM-D-17^PG^ or D-17^PG^/FAM-17′ (5 µL, 250 µM); (c,f) fresh DMEM (240 µL), filtered through 0.45 µm bovine serum albumin (5 µL, 1.5 mM) and FAM-D-17^PG^ or D-17^PG^/FAM-17′ (5 µL, 250 µM). For each condition, three independent transfections were prepared.

### 2.12. Cellular Accumulation Assay

For cellular accumulation assay, 4 h post-transfection cells were washed with PBS to eliminate fluorescently labeled unbound oligonucleotides. Next 24 h, cells were cultivated in full medium. Then, the cells were washed with PBS, trypsinized and suspended in PBS. The accumulation of oligomers was evaluated using NovoCyte (ACEA Biosciences, San Diego, CA, USA) flow cytometer. More than 5000 cells from each sample were analyzed using NovoExpress software (ACEA Biosciences).

### 2.13. Confocal Fluorescence Microscopy

For confocal microscopy, HepG2 cells cultivated in 1 cm^2^ chambers slide (Lab-Tek, Thermo Fisher Scientific Inc., Waltham, MA, USA) were transfected with conjugate FAM-D-17^PG^ or duplex D-17^PG^/FAM-17′ to the final concentration of 5 µM in the media with various composition (see Section 2.11). Right after transfection, cells were washed twice with PBS to eliminate fluorescently labeled unbound oligonucleotides. Washed cell were fixed with 4% paraformaldehyde in DMEM for 30 min and washed twice with PBS. After removing the chamber, the microscopic slide containing cells was mounted with the cover slip glass using ProLong Gold antifade reagent with DAPI (Life Technologies, Eugene, OR, USA), then the slide was kept 24 h in the dark at room temperature. LSM 710 confocal microscope (Carl Zeiss Microscopy GmbH, Jena, Germany) was used in conjunction with Zen imaging software, and images were acquired with a Zeiss 63×/1.40 oil immersion objective. The excitation/emission laser wavelengths were 405 nm (to detect cell nuclei stained with DAPI), 488 nm (to detect FAM).

### 2.14. Statistical Analysis

Each variant of conditions was tested in three or more independent experiments for all investigations. The values reported are expressed as mean ± standard deviation (SD) for at least three independent experiments.

## 3. Results

### 3.1. Oligonucleotides and DOCs in this Study

**Figure 1:**
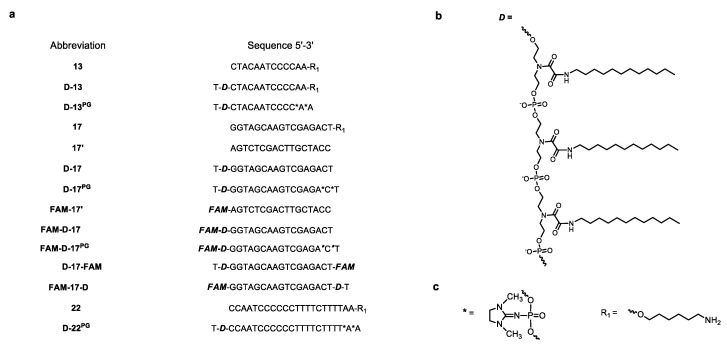
The sequences of oligonucleotides and their conjugates studied in this work (**a**), the structure of “like-a-brush” dodecyl-containing non-nucleoside backbone part of dodecyl oligonucleotide conjugates (DOC) (**b**). All oligonucleotides are deoxy. D-13^PG^, D-17^PG^, FAM-D-17^PG^, D-22^PG^ are oligodeoxynucleotides partially substituted with phosphoryl guanidine groups (PG); * indicated a position of PG modification, R_1_ is the linker with the terminal amino group protecting 3′-end of indicated oligomers from nuclease degradation (**c**). Here, 6-carboxyfluorescein (FAM) is represented 6-carboxyfluorescein residue (See Section 2.3). During post-chromatographic purification, dodecyl-containing oligonucleotides (especially D-17, D-17^PG^, and FAM-D-17^PG^) tended to aggregate, which resulted in significant salting-out of the conjugates at millimolar range concentrations used in the experiments.

In the first step, we designed and synthesized 13-, 17-, and 22-mer oligonucleotide conjugates with three dodecyl chains (Figure 1a). The structures and nucleotide sequences of the oligomers used in this study are given in Figure 1. While planning the synthesis of oligonucleotides with non-nucleoside units bearing dodecyl chains, we considered that the free terminal hydroxyethyl group of this unit can cause degradation of the modified oligonucleotide during the deprotection step in basic aqueous solutions [40,41]. Therefore, we added an extra thymidylate unit at 3′- (FAM-17-D (Figure S3)) or 5′-end (D-13, D-13^PG^, D-17, D-17^PG^, D-17-FAM (Figure S4), D-22^PG^) of corresponding oligomers to overcome this problem. Two phosphoryl guanidine [37,39] (PG) modifications were introduced at 3′-ends of D-13^PG^, D-17^PG^ (Figure S5), and D-22^PG^ oligomers to enhance their stability in serum (Figure 1a,c).

### 3.2. CAC Determination by Nile Red Encapsulation Assay

Further testing by Nile Red encapsulation assay to determine the critical concentration of aggregation for D-17^PG^ partially explained our observations. The Nile Red dye fluoresces intensively in the hydrophobic lipid environment but shows negligible fluorescence in an aqueous medium. Due to these properties, it is applied to determine the LOC’s critical aggregation concentration (CAC) value [42]. The incubation of a certain amount of the dye with D-17^PG^ at varying concentrations revealed an increase in the fluorescence intensity of Nile Red (Figure S7), which evidenced for its encapsulation in a non-polar microenvironment of micellar structure. Control oligonucleotide without dodecyl chains gave no significant increase in Nile Red fluorescence intensity (Figure S7). The CAC value of the DOC micellar particles was extremely sensitive to the presence of magnesium ions. In our work, the CAC of D-17^PG^ conjugate in 15 mM MgCl_2_ (in TAM buffer) was above 1.2 µM (Figure S8a), while without magnesium ions, the CAC value (in TA buffer) was above 25 µM (Figure S8b).

We supposed that the self-assembly of the DOCs occurs with the formation of micellar particles composed of hydrophobic dodecyl inner core and hydrophilic oligonucleotide in the exterior shell corona. Considering this, Mg^2+^ ions can impact the CAC value of DOC micelles by reducing electrostatic repulsion between the oligonucleotides’ charged phosphate groups, stabilizing the micelle structure, and facilitating its formation.

### 3.3. DLS Experiments

**Figure 2:**
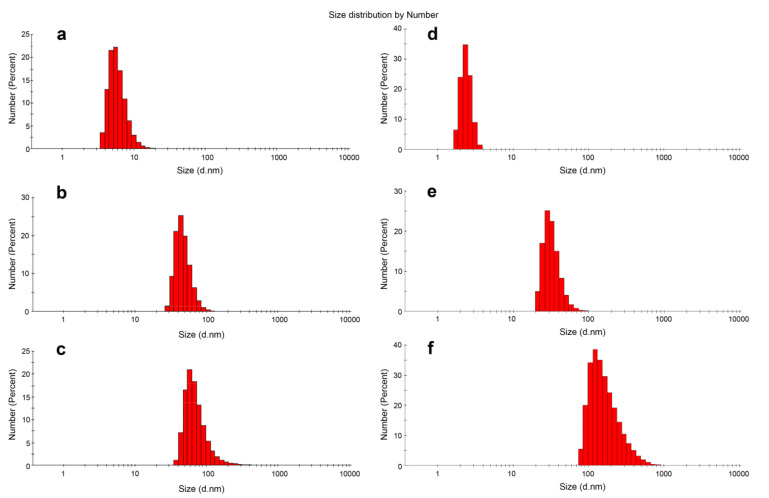
Size distributions of the control oligonucleotides 13 (**a**), 17 (**d**) compared with D-13 (**b**), D-13^PG^ (**c**), D-17^PG^ (**e**) and D-17^PG^ (**f**) conjugates (after 3 h incubation at 5 µM in TAM buffer) as measured by DLS. The D-13^PG^ dodecyl oligonucleotide conjugate with two uncharged PG groups assembled into slightly enlarged micellar particles compared to its DNA analog D-13 (Table 1, Figure 2c). Interestingly, we observed a considerable increase in the size of D-17^PG^ self-assemblies (Table 1, Figure 2f). We attributed these results to the multiple micellar complexes that appeared in a short time in this case, or to more complex micellar structures. Such micellar aggregates of 129.96 ± 73.36 (PDI 0.216) nm in diameter were detected after 24 h incubation for D-17 conjugate.

The size of DOC micellar structures was characterized by DLS measurements. After preincubation in TAM buffer, the average hydrodynamic diameter (D_h_) of D-13 particles was found to be 45.77 ± 13.03 nm (Table 1, Figure 2b), as compared to 5.43 ± 1.60 nm for 13-mer control oligonucleotide without dodecyl groups (Figure 2a). As shown in Figure 2, the assemblies of the DOCs have one peak of populations, which is shifted relative to the control oligonucleotide, and fairly low values of polydispersity indexes (PDI) (Table 1). The intensity distribution also showed one peak of populations with an increase in particle diameter compared to the number mean, which is usually typical for DLS results (Figure S9). Similar results were obtained for longer 17-mer DOC (D-17, Table 1, Figure 2d,e), with the addition of the fact that the D_h_ value of D-17 particles was less than that of D-13 (Table 1).

The average hydrodynamic diameter of D-22^PG^ particles was similar to that of D-13^PG^ assemblies (Table 1). DOC particles formed in solution appear to be much larger than typical NA micelles (up to 10 nm), and their size lies in the range characteristic for vesicle or lamellar structures (up to 500 nm). The non-polar, uncharged PG modification in D-13^PG^ and D-17^PG^ may contribute to forming a more densely packed corona of the oligonucleotide chains around the larger micelle core compared to their deoxy counterparts D-13 and D-17. The results demonstrate no direct correlation between the size of micellar particles and the oligomer length of the DOCs due to the additional impact of the nucleotide sequence, which well correlates with our preliminary DLS studies. We measured hydrodynamic diameters of two additional 22-mer dodecyl conjugates with different heteronucleotide sequences. Micellar particles formed by 5′ D-CTTGACTTTGGGGATTGTAG*G*G 3′ (here D is a three-dodecyl unit and * marks a phosphoryl guanidine modification) and 5′ D-AATACTGCCATTTGTACTG*C*T 3′ conjugates were of 12.15 ± 0.10 and 11.82 ± 1.66 nm correspondingly, while D-22^PG^ formed the particles of increased diameter. The reason for these rather contradictory results remains unclear. We can hypothesize that two additionally studied conjugates can form secondary structures: the sequence of 5′ D-CTTGACTTTGGGGATTGTAG*G*G 3′ contains two G-tracts of 3 and 4 consecutive guanines, which suggests the ability of quadruplex formation, and the sequence of 5′ D-AATACTGCCATTTGTACTG*C*T 3′ may form hairpin and partial self-dimer (Figure S10a). Interestingly, the nucleotide sequence of D-17^PG^ and D-17 also enables a formation of self-dimer of eight nucleotide bases (Figure S10b), which may contribute to the formation of larger particles and/or to accelerate their aggregation. Here, we only briefly discussed the effects of the oligonucleotide length and sequence of the LOC on the micelle size. This issue requires further studies on a wider series of oligonucleotides.

### 3.4. Atomic Force Microscopy

**Figure 3:**
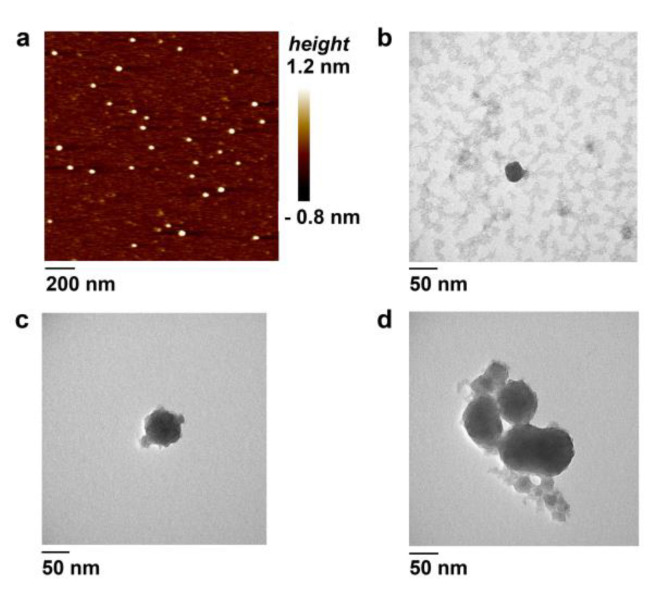
Characterization of D-17 (**a**,**b**) and D-17^PG^ (**c**,**d**) micelles in TAM buffer by atomic force (AFM) (**a**) and transmission electron (TEM) (**b**–**d**), contrasting using uranyl acetate) microscopies. Scale bars are indicated. Each sample contained 1.5 µM (AFM) or 5 µM (TEM) of the corresponding oligomer.

The morphology, dispersity, and relative size of the DOC micelles were investigated by AFM. As shown in Figure 3a, the D-17 sample contains spherical structures with a diameter of 30.5 ± 5.2 nm. The average height of 1.4 ± 0.6 nm for these particles corresponds to those obtained for nucleic acid samples [16,22,23,43]. The average diameter value is in line with our DLS results and correlates with the results of some previous AFM studies of the LOC-formed micellar structures [16,22].

TEM visualization of the D-17 conjugate revealed discrete spherical particles and aggregates (Figure S17) with a size of about 30 nm (Figure 3b). The aggregates represent clusters of individual particles (<10) (Figure S17f). D-17^PG^ particles varied in size from 25 to 50 nm (Figure 3c) and contained more aggregates than the D-17 sample (Figure 3d, Figure S18). This result can also be partly associated with the influence of two uncharged phosphoryl guanidine groups in the sugar-phosphate backbone of D-17^PG^ conjugate, probably facilitating particle aggregation.

In concordance with DLS results, AFM and TEM data indicated that the self-association of DOCs in aqueous solutions leads to the formation of micellar particles with a high tendency to aggregate.

### 3.5. Electrophoretic Mobility Shift Assay

**Figure 4:**
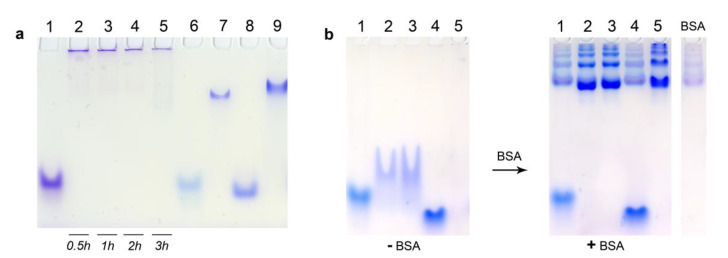
Comparative electrophoretic mobilities of control oligonucleotides and dodecyl oligonucleotide conjugates. (**a**) 17 (Lane 1), D-17^PG^* (Lanes 2–5), 13 (Lane 6), D-13^PG^ (Lane 7), 22 (Lane 8), D-22^PG^ (Lane 9) investigated by electrophoretic mobility shift assay (EMSA) in non-denaturing 6.5% PAAG after 3 h* incubation in TAM buffer, at 25 °C; each sample contained 20 µM of the oligomer. (**b**) 13 (Lane 1), D-13 (Lane 2), D-13^PG^ (Lane 3), 17 (Lane 4), D-17^PG^ (Lane 5) investigated by non-denaturing 8% PAGE after 3 h incubation at 35 °C and additional no (−BSA) or adding (+BSA) of 45 µM bovine serum albumin (BSA without oligomers control lane is depicted on the right); each sample contained 25 µM of the oligomer in TAN buffer. Bands were visualized by Stains-All staining. * This conjugate was loaded into the wells after the indicated time of incubation.

The investigation of DOCs self-assembly by EMSA also showed results well-consistent with the DLS data. As shown in Figure 4a, the D-17^PG^ sample forms extremely large structures (or aggregates), which cannot move along the gel and remain close to wells’ bottoms (Figure 4a, Lanes 2–5). At the same time, micellar D-13^PG^ particles demonstrated higher electrophoretic mobility than D-22^PG^ (Figure 4a, Lanes 7 and 9 correspondingly). Analysis of the electrophoretic mobility of D-17^PG^ at the indicated time of incubation demonstrated that low-mobility structures form in as little as 30 min (Figure 4a, Lane 2). All control oligonucleotides without dodecyl chains were characterized by significantly higher electrophoretic mobilities as compared to the conjugates (Figure 4a, Lanes 1, 6, 8).

Next, we compared the affinity of DOCs and dodecyl-free oligonucleotides to serum albumin. Having confirmed the formation of DOC micelles by D-13, D-13^PG^, and D-17^PG^ conjugates in conditions similar to physiologic (Figure 4b, (−BSA), Lanes 2,3,5), we added the bovine serum albumin (BSA) and allowed the reaction mixture to equilibrate for 30 min. After incubation with BSA (1.8 molar excess relative to the oligomer), the dodecyl-containing conjugates completely bound to albumin in contrast to the parent control oligonucleotides 13, 17 (Figure 4b, ((+BSA), Lanes 2,3,5).

Albumin possesses seven major binding sites for fatty acids with high and moderate affinity [44]. Interestingly, we have demonstrated that D-13^PG^ DOC is highly associated with BSA even at the 0.1–0.15 molar excess (that is, the lack) of the protein (Figure S19). This corresponds to the interaction of one albumin molecule with approximately ten DOC molecules. These results point that in blood serum with high levels of albumin (about 0.5–0.6 mM), DOCs would be most probably entirely bound by this protein.

**Figure 5:**
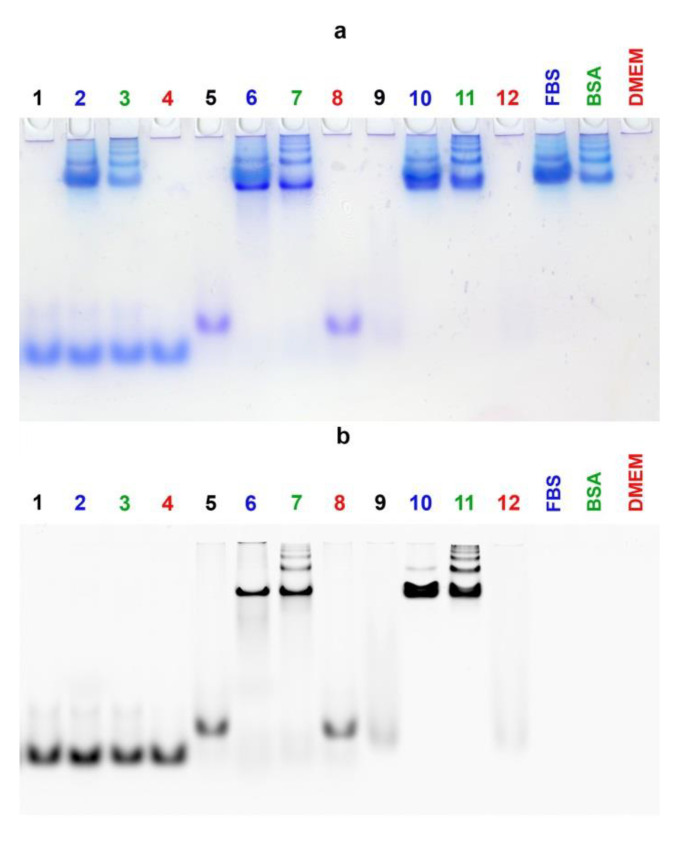
Comparative electrophoretic mobilities of 5 µM control duplex FAM-17′/17 (Lanes 1–4), dodecyl-containing duplex FAM-17′/D-17^PG^ (Lanes 5–8) and the FAM-D-17^PG^ conjugate (Lanes 9–12) investigated by EMSA in non-denaturing 8% PAAG after 2 h incubation at 37 °C in DMEM medium (depicted in red), DMEM supplemented with 30 µM BSA (depicted in green), DMEM supplemented with 10% fetal bovine serum (FBS) (depicted in blue). All medium conditions without oligomers control lanes are depicted on the right in corresponding colors. Indicated oligomers after incubation in PBS are depicted in black for additional controls. Bands were visualized after electrophoresis by Stains-All staining (**a**) and by recording the image after scanning with laser excitation at 488 nm (**b**). It is interesting to note that during the electrophoretic analysis of micellar assemblies of FAM-labeled DOCs, we observed significant fluorescence quenching of their bands, in contrast with the bands of non-conjugated control oligomers (Figure 5, Lanes 9, 12, Figure S20).

As expected, the EMSA (Figure 5) proved the complete association with the proteins either for FAM-17′/D-17^PG^ duplex (Lanes 6,7) or FAM-D-17^PG^ conjugate (Lanes 10,11) in the medium supplemented with BSA, as well as with 10% FBS. The FAM-17′/17 control duplex remained unbound in this assay (Lanes 2–3).

### 3.6. Fluorescence Quenching Experiments

The dyes in the vicinity of micellar arrangements can displace a complex physicochemical and photophysical behavior. Some studies evidenced that the fluorescence intensity of fluorescein derivatives may depend not only on the pH value [45], but also on the complicated intermolecular, hydrophobic/electrostatic interactions between the fluorophore, and the lipids in the microenvironment. It is important to gain insights into these non-specific interactions of the LOCs with the dyes that are widely used to detect intracellular localization of NA-based constructs. The fluorescence quenching of some dyes can be induced by dynamic (or collisional), static, or combined dynamic and static complex mechanisms. Self-quenching and fluorescence resonance energy transfer (FRET) can often occur, due to molecular interactions between the molecules of fluorophores themselves in proximity to each other [46].

**Figure 6:**
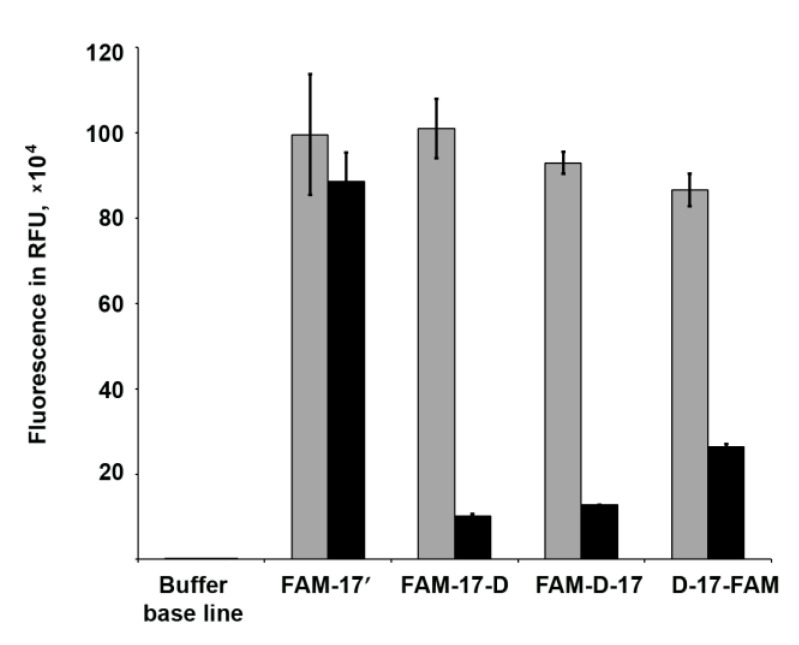
Fluorescence intensity values of 1.5 µM fluorescently labeled FAM-17′, FAM-17-D, FAM-D-17, and D-17-FAM oligomers in TAM (black columns) or TA (grey columns) buffer conditions. For excitation and emission scanning parameters, see Section 2.9. We hypothesized that during the FAM-D-17 self-assembly, FAM residues are encapsulated into the micelle core, thereby interacting with each other in close proximity and providing significant fluorescence quenching. For instance, fluorescence from Oregon green residues in dye-conjugated titin molecules quenched in the native folded state of the protein due to the proximity of dye residues [47].

To explore if self-assembly affects the fluorescence of 6-carboxyfluorescein (FAM) residue combined with DOCs, we investigated fluorescence intensities (FI) and absorption spectra of FAM-labeled oligomers with dodecyl groups. Dodecyl-free oligonucleotide FAM-17′ served as a control. The obtained results evidence for 86–87% fluorescence quenching of FAM-D-17 under the conditions of micelle formation in TAM buffer, in comparison with FI value in TA buffer lacking Mg^2+^, where 17-mer DOC did not reach CAC at the used concentration (Figure 6). At the same time, in the case of dye-labeled control oligonucleotide FAM-17′, the addition of Mg^2+^ did not affect the fluorescence intensity (Figure 6).

Further, we compared the FI values of FAM-17-D and D-17-FAM conjugates in the presence/absence of magnesium ions. These DOCs bear dodecyl chains and the FAM residue at the opposite ends of the 17-mer oligonucleotide, which is assumed to exclude dye encapsulation in the micellar inner core. We supposed that if interactions inside the core cause the fluorescence decrease, these conjugates will demonstrate the smaller quenching degree. Interestingly, the fluorescence intensity of the D-17-FAM, as expected, was quenched less (by 67–72%), while the FI value of the other conjugate FAM-17-D with 5′-terminal dye residue surprisingly decreased by 89–91%. We attribute this difference to the existence of multiple mechanisms of molecular interactions involved in fluorescence quenching. Fluorescence of fluorescein and its derivatives are extremely sensitive for the quenching, even to the type of the binding linker [48]. An earlier finding of fluorescence quenching of 6-carboxyfluorescein in liposomes was reported decades ago [49]. The authors concluded that dimerization of the dye and energy transfer to nonfluorescent dimers made a major contribution to the mechanisms of this concentration quenching phenomenon. The absorbance spectra of FAM-D-17, FAM-17-D, and D-17-FAM DOCs also contain shoulders at about 468 nm (Figure S21). Furthermore, these spectra show differences in absorbance of the conjugates in solutions with or without Mg^2+^ (Figure S21), so a possibility of ground-state complex formation also cannot be excluded. It has been reported that Mg^2+^ ions up to 15 mM do not cause appreciable fluorescence quenching of 5′-fluorescein-labeled DNA oligonucleotides [50]. On the other hand, 5-FAM and TAMRA-modified oligonucleotides significant fluorescence quenching during hybridization with a complementary strand is caused by photoinduced electron transfer between the fluorophore and nucleotide base [51]. It has been revealed that guanine bases can strongly quench the fluorescence of the dyes mentioned above. The presence of two guanines at 5′-end of the FAM-D-17 and FAM-17-D DOCs can add one more step to complex molecular interactions of quenching mechanisms. These underlying diverse and probe-dependent mechanisms are still exactly unknown and require careful use of fluorophores in LOC-based systems.

### 3.7. Cellular Uptake of Three Dodecyl-Containing Conjugates

**Figure 7:**
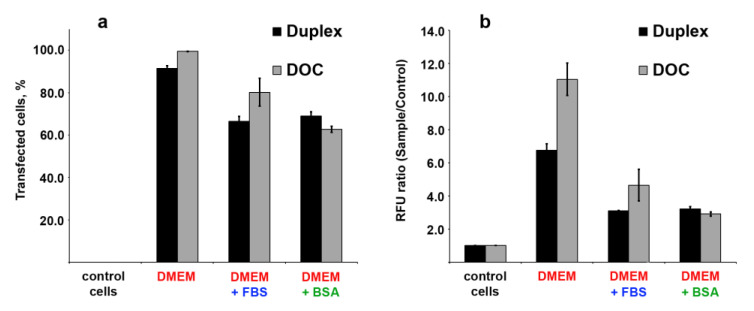
Cellular uptake of fluorescently labelled DOC (FAM-D-17^PG^) and its duplex (D-17^PG^/FAM-17′) estimated by flow cytometry after 4 h incubation of HepG2 cells with conjugates and additional followed 14 h without it: (**a**) percentage of FAM-positive HepG2 cells in the population; (**b**) normalized median value of the cell fluorescence to the autofluorescence of control cells. The highest transfection efficacy was achieved for cells treated with FAM-D-17^PG^ in DMEM (Figure 7a,b). All cells were transfected (Figure 7a), and the median value of their fluorescence intensity exceeded that of cells’ auto-fluorescence 11 ± 1 times (Figure 7b). High efficacy of transfection was also reached for cells transfected with duplex D-17^PG^/FAM-17′ in DMEM (Figure 7a). Surprisingly, the median value of cells’ fluorescence was almost two times lower than for cells treated with a single stranded dodecyl-containing oligonucleotide (Figure 7b).

We also examined the cellular accumulation of FAM-labeled lipophilic oligonucleotide conjugates bearing three dodecyl groups (FAM-D-17^PG^) or their duplex (D-17^PG^/FAM-17′). The concentration of oligonucleotides was determined by CAC to ensure that oligonucleotide derivatives present in an aggregated state in the transfection solution. As shown above, the addition of BSA or FBS to the DOC aggregates leads to the protein binding of the lipophilic conjugate (Figure 5). To define the influence of BSA or other serum proteins on cellular internalization of DOCs, the transfection of HepG2 cells was performed with 5 μM concentrations of conjugated oligonucleotide in DMEM, either alone or supplemented with 10% FBS or 30 μM BSA. Prior to transfection procedure, we verified the stability of FAM-D-17^PG^ conjugate and D-17^PG^/FAM-17′ duplex in 10% FBS (Figure S22). The efficacy of transfection was evaluated using flow cytofluorometry. The results demonstrated that DOCs penetrate the human cells with different efficacy, depending on the medium (Figure 7).

We attributed the difference in cells’ fluorescence intensity to the different numbers of fluorescent groups in the aggregates. The formation of micellar structures of similar size with oligonucleotides exposed on surface and dodecyl groups forming a hydrophobic core requires a comparable number of oligonucleotide molecules. This comes from the fact that the size of oligonucleotide or duplex determines the curvature of the particle’s surface and, therefore, its diameter. However, in the duplex, only one strand bears a fluorescent group, so the aggregate formed by a duplex with a diameter of 85.4 ± 17.0 nm (DLS data in PBS) contain twice less FAM groups than the aggregate formed by single strand conjugate with a diameter of 96.9 ± 47.8 nm.

The presence of BSA or BSA-containing FBS in the transfection media decreases the transfection efficacy (Figure 7a) and the accumulation of the oligonucleotide cargo in the cell (Figure 7b). We explain this result by the fact that aggregates formed by both oligonucleotides conjugated with three dodecyl groups and their duplexes appear in the BSA/DOCs associates (Figure 5, Lanes 7,11). The obtained data suggest that binding to albumin inhibits DOC’s cell penetration since accumulation rates of oligonucleotide-albumin complexes are lower than those for aggregates or linear nucleic acids. Surprisingly, the efficacy of FAM-D-17^PG^ accumulation in cells in the presence of FBS is higher than in the presence of BSA only (Figure 7a). This difference can be explained by the presence of other lipids in FBS, including fatty acids, which can compete with conjugate’s dodecyl groups for interaction with albumin [34,44,52]. In this case, the fraction of unbound FAM-D-17^PG^ released from protein/DOCs associates can penetrate cells. At the same time, D-17^PG^/FAM-17′ duplex in the presence of FBS demonstrated results comparable to those for BSA containing transfection. The reason for the difference between single stranded molecules of FAM-D-17^PG^ and D-17^PG^/FAM-17′ duplexes in this context is a larger negative net charge of the duplex [33].

**Figure 8:**
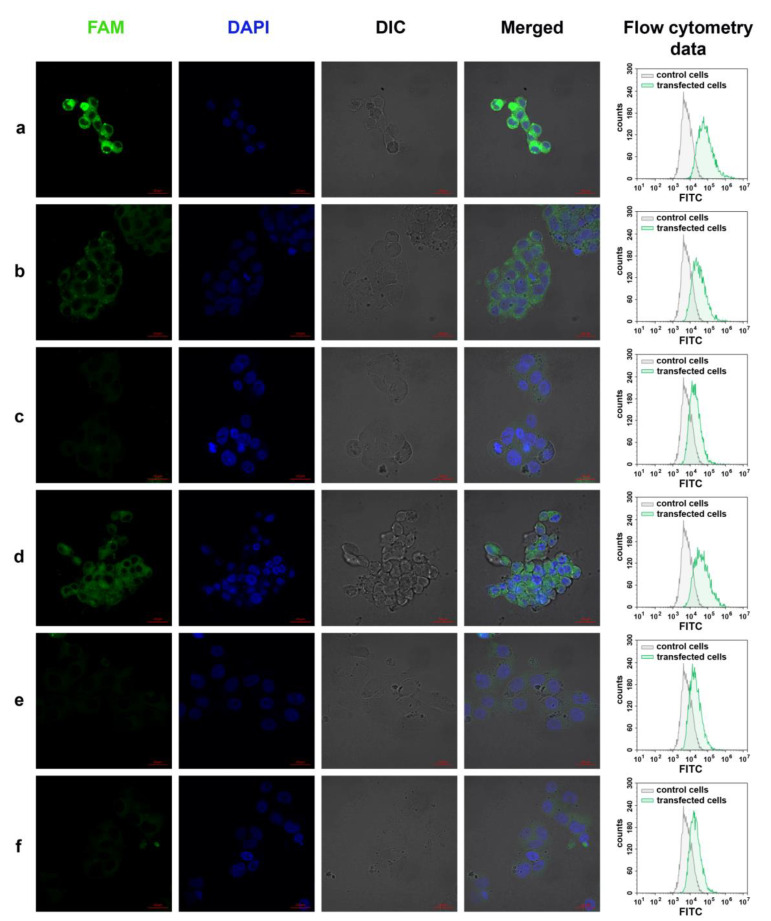
Qualitative (confocal fluorescent microscopy imaging) and quantitative (flow cytometry data) characterizations of HepG2 cells treated with 5 µM fluorescently labeled either FAM-D-17^PG^ (**a**–**c**) DOC or D-17^PG^/FAM-17′ (**d**–**f**) duplex in DMEM medium (**a**,**d**); in DMEM supplemented with 10% FBS (**b**,**e**); in DMEM supplemented with 30 µM BSA (**c**,**f**). The red bar in confocal fluorescent images corresponds to 20 µm. Our experiments are consistent with a previous study reporting micelle formation for other DNA-based amphiphilic conjugates depended on the presence of Mg^2+^ [15,16]. In another study, the authors also suggested that Mg^2+^ stabilizes all the lipid-oligonucleotides micellar assemblies, probably due to the Mg^2+^-mediated neutralization of the oligonucleotide negative charges [53].

The most intensive fluorescence was registered for the cells treated with DMEM-diluted FAM-D-17^PG^ or D-17^PG^/FAM-17′ (Figure 8a,d). Fluorescent signals of the medium intensity were found in cells transfected with FAM-D-17^PG^ dissolved in DMEM supplemented with 10% FBS (Figure 8b); for other samples, FAM signals were too low (Figure 8c,e,f). In FAM-positive cells, a fluorescent signal was evenly distributed throughout the cytoplasm, and no co-localization with nuclei was revealed.

These results indicate that oligonucleotides conjugated with three dodecyl groups can penetrate cultured human cells both as disassembled molecules and in aggregated form.

## 4. Discussion

In recent years, several approaches have been proposed to employ “like-a-brush” LOCs as useful tools in the field of NA-based formulations [10,14,18,22,33,35,53,54,55,56]. Most of them rely on the conjugates bearing the diacyl lipid group. A few data have been reported on the features of triple lipophilic chains-tethered oligonucleotides [14,33]. Their investigations are generally limited by high hydrophobicity, fast self-aggregation, and salting out in aqueous solution. So far, only one paper reports two 15-mer oligoadenylate and oligothymidylate LOCs containing three “like-a-brush” hydrophobic chains [14]. The particle size and shape for the latter conjugate were characterized by DLS, TEM, and scanning electron microscopy, while highly hydrophobic oligoadenylate LOC irreversibly self-aggregated immediately after the deblocking procedure [14].

The present research aimed to investigate 13-, 17-, and 22-mer “like-a-brush” triple chains-contained DOCs for their self-assembly features and the ability to enter the cells. We have chosen heteronucleotide sequences for these oligomers and inserted two phosphoryl guanidine modifications at 3′-end of some conjugates during standard phosphoramidite solid-phase synthesis to provide additional nuclease stability [37,39]. Due to high hydrophobicity, DOCs form micellar particles in micromolar concentrations and require extreme caution to avoid their salting out during the work.

We observed a larger size of D-13 and D-17 DOC particles as compared with another 15-mer oligothymidylate LOC, also containing “like-a-brush” hydrophobic moiety with three aliphatic tails [14]. In turn, the conjugates containing phosphoryl guanidine modifications D-13^PG^, D-17^PG^, D-22^PG^ formed larger micellar assemblies than their DNA counterparts. There is a little discussion in the literature that tends to focus on the oligonucleotide length, sequence, and modified sugar-phosphate backbone of the LOC regarding the size of a micelle formed. The research also reported various sizes of LOC self-assemblies, which demonstrated the required biological effect. The impact of nucleotide sequence on LOC micellar assemblies’ size was described for two 19-mer PS conjugates bearing identical diacyl lipid groups at their 5′-ends. While the oligomer 5′-AACTTGTTTCCTGCAGGTGA-3′ formed small particles of ~11 nm, 5′-CGTGTAGGTACGGCAGATC-3′ yielded micellar structures with the size of more than 100 nm [10]. Recently it was shown [11] that BODIPY-conjugated 10- and 25-mer oligodeoxythymidylates self-aggregate with a formation of the same assemblies of 94.1 ± 20.4 and 75.5 ± 4.4 nm, correspondingly. Self-association of siRNA with one BODIPY-attached strand into nanosized aggregates of ~140 nm in aqueous solution was shown to be indispensable for the high cellular uptake of these duplexes and efficient gene regulation by RNA interference [11]. The siRNA-squalene conjugates also demonstrated self-organizing in water with the formation of ~165 nm particles, and after intravenous injections, inhibited tumor growth in a mice xenograft model of papillary thyroid carcinoma [21]. Most reports deal with the development of LOC-based constructions for anticancer therapy [10,13,21,23,24,29,35]. Passive targeting via enhanced permeability and retention (EPR) effect requires a 5–200 nm hydrodynamic size range for the formulations [57,58]. Therefore, the ability of DOCs to self-assembly into micellar particles and their aggregates of 30–170 nm in size seems to open the way for further in vitro and in vivo studies to examine their cell penetration efficacy.

Given the high plasma concentration of albumin in vivo, we evaluated the affinity of DOCs for association with BSA. Other findings confirm our BSA binding experiments. It was reported that approximately 93% of LOC micelles dissociated into a non-micellar state when incubated in vitro with 0.1 mM BSA [22]. Size exclusion chromatography method has shown that diacyl lipid conjugated PS oligonucleotide in aqueous solution elutes as micelles, but after the following incubation with FBS, nearly 50% of this conjugate co-migrate with the albumin fraction [54]. In another work, siRNA bearing short-chain fatty acid residues such as lauroyl did not bind to lipoproteins in vivo, either associated with serum albumin or remained unbound [26]. Interestingly, monododecyl-containing siRNA bound to albumin with the highest affinity (K_d_ ~200 µM) among other investigated lipophilic residues, and the protein was saturated with these conjugates at 1:3.6 molar ratio [26].

The readily occurred interaction of DOCs with albumin can increase their overall in vivo lifetime [5]. An analogy can be drawn with recent reviews regarding more extensively studied interaction of PS modified therapeutics with proteins [59,60]. Oligonucleotides modified in PS backbone bind to a number of plasma proteins, including albumin. Plasma proteins binding increase the circulation half-life of PS oligomers which is crucial to maintain their distribution to peripheral tissues [60].

On the other hand, a well-known criticism of increased affinity of LOCs for binding by albumin refers to observations of subsequent conjugates accumulation in the liver and lymph nodes than in other organs [54,61]. To find a possible solution to this challenge, an interesting experiment was carried out with stable G-quadruplex-locked DNA micelles, which could not associate with serum albumin [22]. Cellular uptake of these conjugates was negligible in comparison with DNA micelles without intermolecular G-quadruplexes, which retain the ability to albumin bind [22].

In contrast to this limitation, other studies established albumin as a drug carrier [62]. Of particular interest are several features of albumin, which are responsible for the accumulation of this plasma protein in solid tumors [34,63]. The Paclitaxel albumin-bound particles known as Abraxane^®^ are used in cancer therapy [62]. Recent research [35] suggested using in situ albumin targeting for development of carrier-free RNAi-based cancer therapies. The synthesized siRNA conjugated to a diacyl lipid moiety, which rapidly binds albumin in situ, was shown to achieve 19-fold greater tumor accumulation and 46-fold increase in per-tumor-cell uptake in a mouse orthotopic model of human triple-negative breast cancer as well as elicits sustained silencing in an in vivo tumor model [35]. The tumor:liver accumulation ratio of more than 40:1 achieved by this diacyl lipid-tethered siRNA is a promising result for LOC-based formulations. Future studies in this area are therefore required more attention to design of the research. Although most LOCs bind to albumin with high affinity, studies reporting the effect on intracellular uptake during the transfection are scarce. Recently it was found that BSA treatment reduces cell penetration of PS ASOs and their lipid conjugates in a protein concentration-dependent manner [25]. Interestingly, the cellular uptake efficacy of the lipid-conjugated PS ASO was reduced much stronger than that of the parental unconjugated oligonucleotide. Almost all other articles describe cells in vitro transfection in the absence of serum and/or albumin in the media [10,11,21,22,25], although understanding the features of interactions between LOCs and proteins is crucial for their use in vivo.

Taken together, in this study, we obtained key results proving that DOCs represent the attractive objects for further design of transport systems in oligonucleotide-based therapeutics. For future in vivo research of the DOCs, a principal issue is to evaluate the importance of the ability to form spherical micellar particles in aqueous media in front of their high protein binding affinity.

## 5. Conclusions

To summarize, we designed and conveniently synthesized DOCs containing three “like-a-brush” lipophilic chains. Phosphoryl guanidine modifications were introduced at the 3′ end of some DOCs to prolong the plasma exposure. In the current work, we present the investigation of self-assembling and cell-penetrating features of these conjugates. DLS, AFM, and TEM techniques showed self-association of dodecyl oligonucleotide conjugates into spherical micellar particles and their nanosized aggregates (<200 nm). These structures are highly bound by serum albumin, which can increase their circulation half-life and bioavailability. It was found that DOCs and their duplexes can penetrate HepG2 cells by mimicking the spherical architecture or anchoring nonspecifically to the membranes both in the absence (with high efficacy) and in the presence of serum albumin (with reduced efficacy). These results, along with recently obtained data [33] indicate a strong potential to consider these conjugates as essential nanomaterials to develop nucleic acid delivery tools for biomedical applications.

## Figures and Tables

**Table 1:** The average hydrodynamic diameter (D_h_) of the DOC micellar particles.

DOC Type	D_h_^1^, nm	PDI
**D-13**	45.77 ± 13.03	0.109
**D-13^PG^**	65.30 ± 33.66	0.241
**D-17**	32.67 ± 9.07	0.214
**D-17^PG^**	169.63 ± 96.40	0.225
**D-22^PG^**	80.76 ± 31.33	0.148

^1^ as calculated by dynamic light scattering (DLS) measurements after 3 h incubation of 5 µM DOC in TAM buffer.

## Supplementary Materials

The following are available online at https://www.mdpi.com/2079-4991/10/10/1948/s1, Figure S1: The structure of 3′-FAM CPG used in this work, Figure S2: Comparative electrophoretic mobilities of oligonucleotides and DOCs, Figures S3–S5: The structures of FAM-17-D, D-17-FAM and FAM-D-17^PG^ conjugates, Figure S6: Typical MALDI TOF and ESI mass spectra of the conjugates, Figure S7,S8: Self-assembly of 17-mer DOC by Nile Red binding assay, Figure S9: Dynamic light scattering measurements, Figure S10: Possible structures of DOCs self-dimers, Figures S11–S16: AFM additional images, Figures S17,S18: TEM additional images, Figure S19: BSA binding with D-13^PG^ conjugate, Figure S20: Fluorescence quenching additional image, Figure S21: Absorbance spectra of FAM-D-17, FAM-17-D, D-17-FAM, Figure S22: The stability of FAM-D-17^PG^ conjugate and FAM-17′/D-17^PG^ duplex used for transfection in 10% FBS, Table S1: Experimental and theoretical molecular masses of the DOCs.
