# Supplementary material for: Amphiphilic “Like-A-Brush” Oligonucleotide Conjugates with Three Dodecyl Chains: Self-Assembly Features of Novel Scaffold Compounds for Nucleic Acids Delivery
Source: Nanomaterials (Basel). 2020 Sep 29;10(10):1948. doi: 10.3390/nano10101948 (PMC7600535; doi:10.3390/nano10101948)
Supplement: Supplementary file 1 [file nanomaterials-10-01948-s001.pdf]

Supplementary Materials: Amphiphilic "Like-a-Brush" Oligonucleotide Conjugates with Three Dodecyl Chains: Self-Assembly Features of Novel Framework Compounds for Nucleic Acids Delivery

Anna S. Pavlova, Ilya S. Dovydenko, Maxim S. Kupryushkin, Alina E. Grigor'eva, Inna A. Pyshnaya and Dmitrii V. Pyshnyi *

Contents

Abbreviations
S1. Post-synthetic separation and purification of oligomers and DOCs ……………………2
S1. a. Figure S1. The structure of 3′-FAM-CPG ………………………….…………5
S1. b. Oligonucleotide homogeneity by denaturing electrophoresis (Figure S2)…...6
S1. c. Figure S3. The structure of FAM-17-D conjugate …………………….…....…..7
S1. d. Figure S4. The structure of D-17-FAM conjugate …………..………….......…..8
S1. e. Figure S5. The structure of FAM-D-17^{PG} conjugate ………………….......…..9
S1. f. Table S1. Experimental and theoretical molecular masses of the DOCs …....10
S1. g. Figure S6. Typical MALDI TOF and ESI mass spectra of the conjugates …..11
S2. Figures S7-S8. Self-assembly of 17-mer DOC by Nile Red binding assay ………..12-13
S3. Figure S9. Dynamic light scattering measurements ……………………..…………...14
S4. Figure S10. Possible structures of DOCs self-dimers …………….....…………………..15
S5. Figure S11-S16. AFM additional images…….……..……………………………...…..16
S6. Figures S17-S18. TEM additional images …………………...………….…………….…22
S7. Figure S19. BSA binding with D-13^{PG} conjugate ……………………………………..…24
S8. Figure S20. Fluorescence quenching phenomenon ……………………..……………25
S9. Figure S21. Absorbance spectra of FAM-D-17, FAM-17-D, D-17-FAM ……….......26
S10. Figure S22. The stability of FAM-D-17^{PG} conjugate and FAM-17′/D-17^{PG} duplex used for transfection in 10% FBS ……………………………………………………………………….27

Abbreviations

DOC—dodecyl oligonucleotide conjugate

D—here represents a non-nucleosidic unit bearing three dodecyl chains

FAM—here represents the residue of 6-carboxyfluorescein

BSA—bovine serum albumin

FBS—fetal bovine serum

DMEM—Dulbecco's Modified Eagle Medium

PAGE—polyacrylamide gel electrophoresis

PAAG—polyacrylamide gel

HPLC—high pressure liquid chromatography

CAC—critical aggregation concentration

XC—xylene cyanol FF loading dye

BP—bromophenol blue loading dye

Abbreviations of olginucleotides and their conjugates are used in accordance with the main text of the article.

S1. Post-synthetic separation and purification of oligomers and DOCs

Native and modified oligonucleotides were isolated by reverse-phased HPLC on an Agilent 1200 HPLC system (Agilent Technologies, Santa Clara, CA, USA) using a Zorbax SB-C18 5 mm column 4.6 × 150 mm in a linear gradient of acetonitrile (0–50% or 0–90%) in 20 mM triethylammonium acetate, pH 7.0, at flow rate of 2 mL min^{-1}. Fractions containing the desired product were pooled, concentrated in vacuo, dissolved in 0.1 mL of deionized water, and precipitated by 1ml of 2% $LiClO_4$ (or $NaClO_4$) in acetone. After centrifugation at 14500 rpm for 2 min, washing with acetone and drying on air for 20 min, oligonucleotide pellets were dissolved in deionized water and stored at −20°C. Oligonucleotides homogeneity was assessed by electrophoresis in 15% denaturing PAAG with 8 M urea, in 0.089 M Tris-Borate, pH 8.3, as a running buffer. The oligonucleotide bands were visualized in gel by Stains-All staining or for FAM-labeled in addition by scanning and recording the image using VersaDocTM MP 4000 Molecular Imager® System (Bio-Rad, Hercules, CA, USA) after excitation at 488 nm.

Molecular masses of the oligonucleotides and conjugates (for examples of the conjugates structures see Figures S3–S5) were confirmed by ESI or MALDI TOF mass spectrometry. ESI mass spectra recorded on an Agilent G6410A LC-MS/MS triple quadrupole ESI mass spectrometer (Agilent Technologies, Santa Clara, CA, USA) in the MS scan mode with negative ion detection. The

samples were dissolved to 0.1 mM concentration in 20 mM triethylammonium acetate containing 60% acetonitrile for direct injection (10 μl). MALDI TOF mass spectra recorded using Reflex III, Autoflex Speed (Bruker, Germany) with 3-hydroxypicolinic acid (HPA) as a matrix in a positive ion mode. Experimental molecular masses were calculated using m/z values, obtained for each sample from mass spectrum (Table S1, for examples of spectra see Figure S6).

S1. a. The structure of 3′-FAM-CPG used in this work

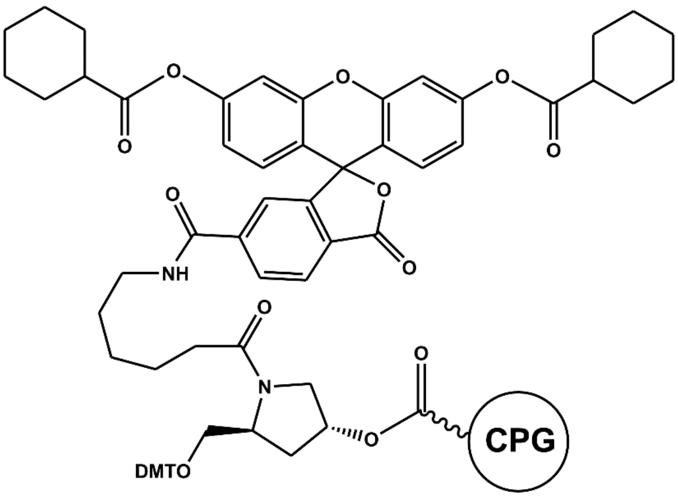

Figure S1. The structure of 3′-FAM-CPG used in this work to synthesize D-17-FAM conjugate.

S1. b. Oligonucleotide homogeneity by denaturing electrophoresis

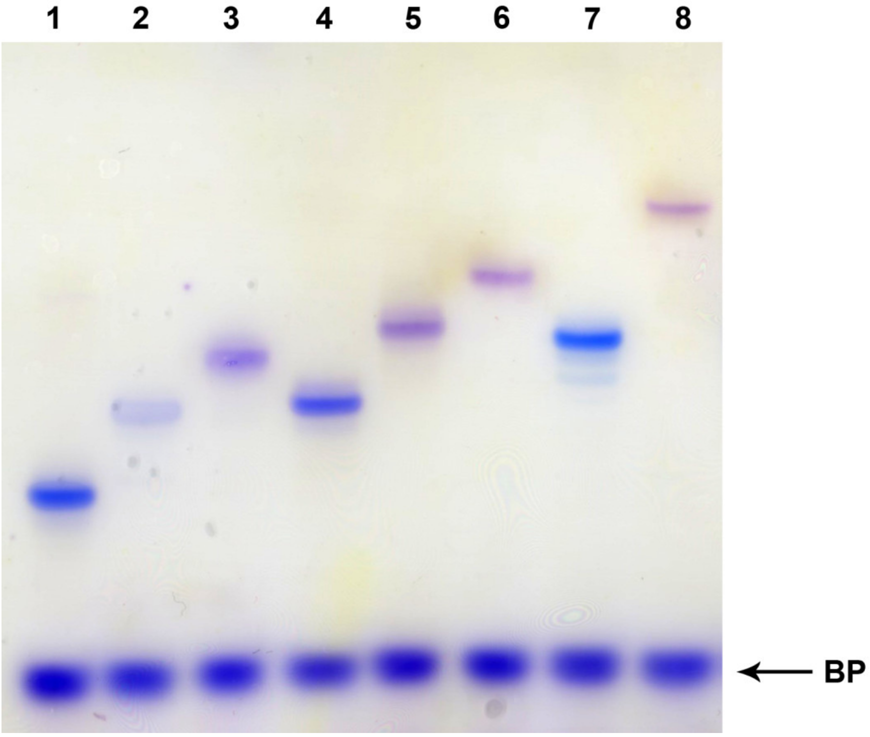

Figure S2. Comparative electrophoretic mobilities of oligonucleotides 13 (Lane 1), 17 (Lane 4), 22 (Lane 7) and dodecyl oligonucleotide conjugates D-13 (Lane 2), D-13^{PG} (Lane 3), D-17 (Lane 5), D-17^{PG} (Lane 6), D-22^{PG} (Lane 8) investigated by denaturing electrophoresis in 15% PAAG (8 M urea, 0.089 M Tris-Borate, pH 8.3). Bands were visualized by Stains-All staining. BP is a lead dye.

S1. c. Figure S3. The structure of FAM-17-D conjugate

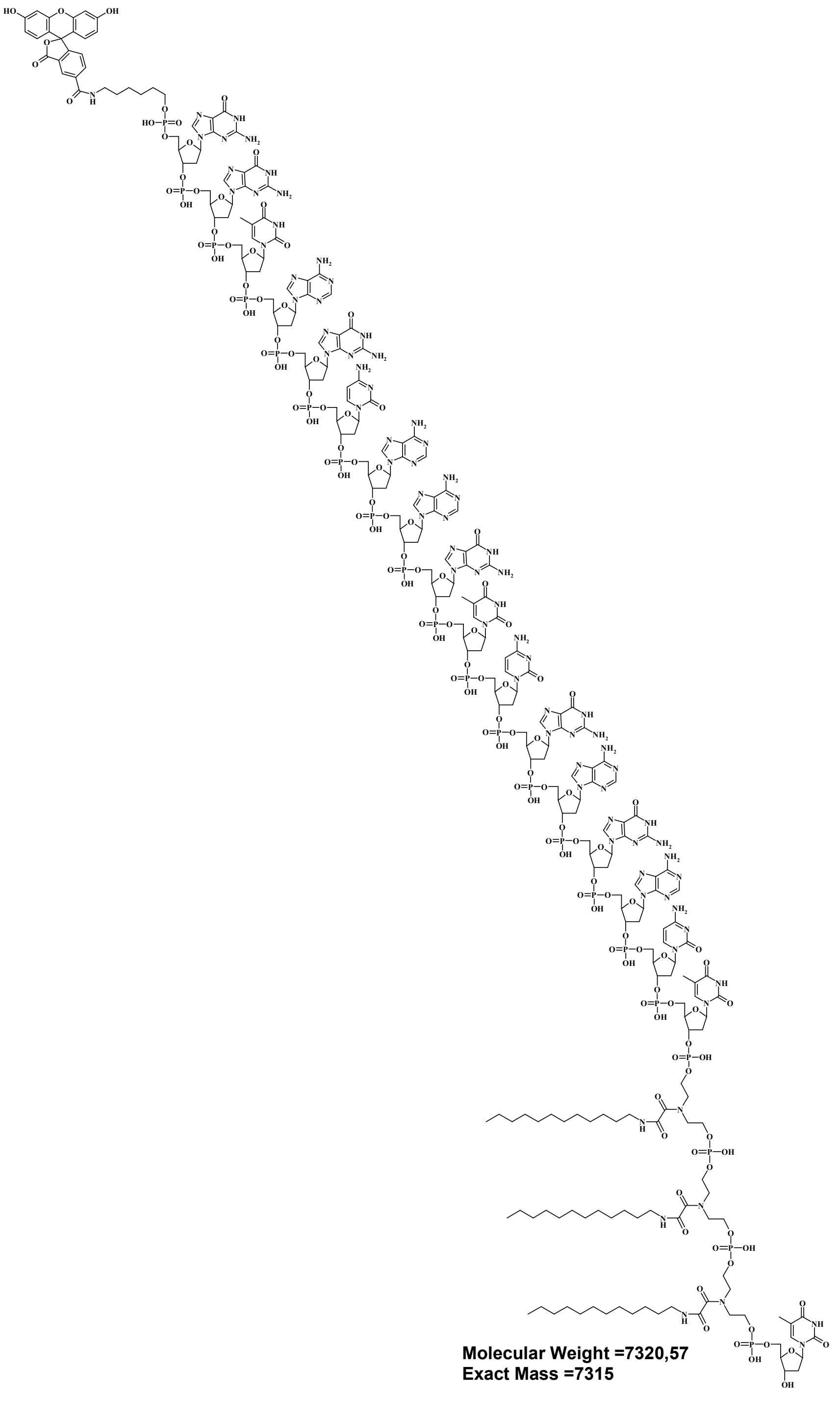

Figure S3. The structure of FAM-17-D conjugate.

S1. d. Figure S4. The structure of D-17-FAM conjugate

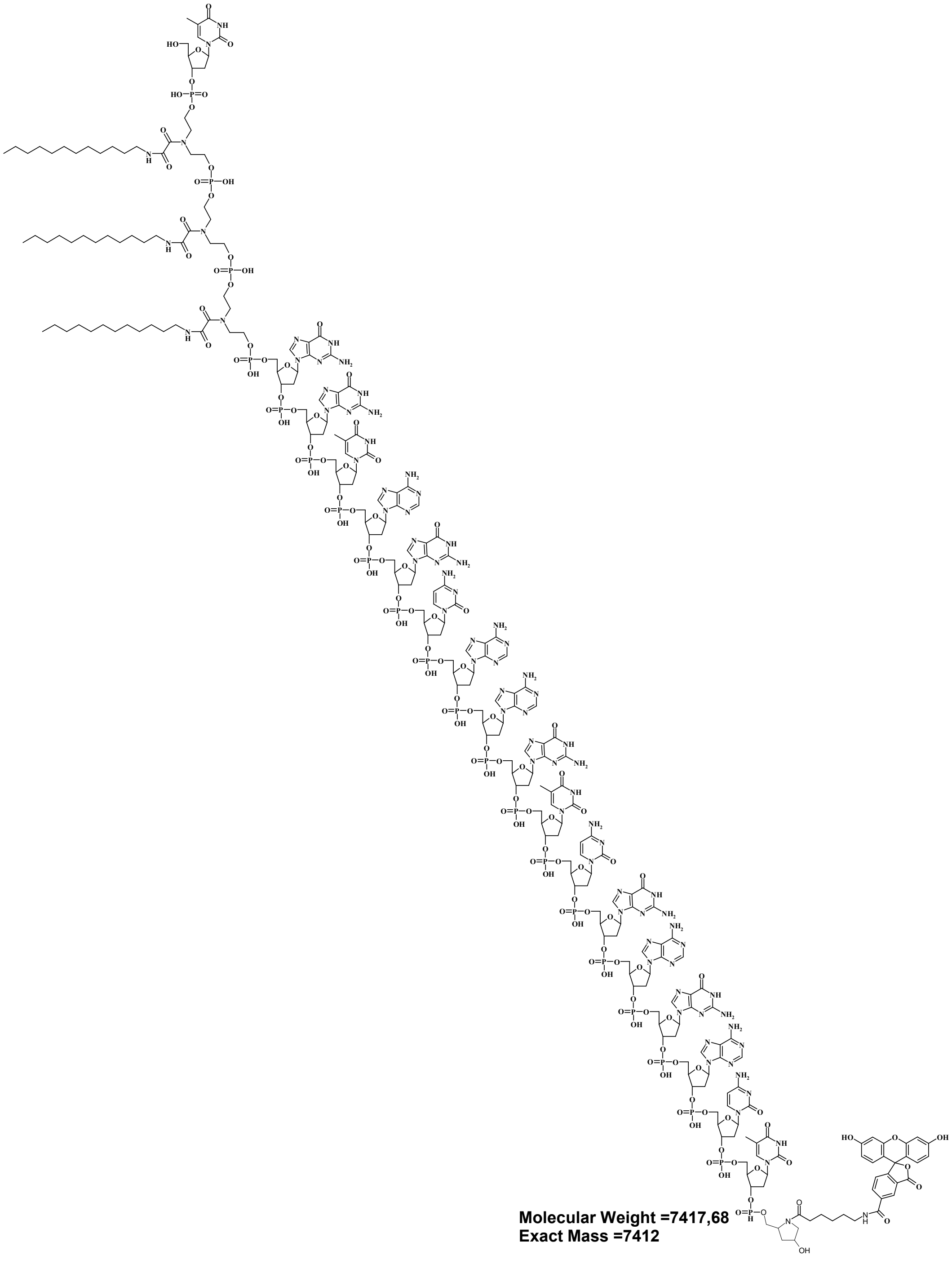

Figure S4. The structure of D-17-FAM conjugate.

S1. e. Figure S5. The structure of FAM-D-17^{PG} conjugate

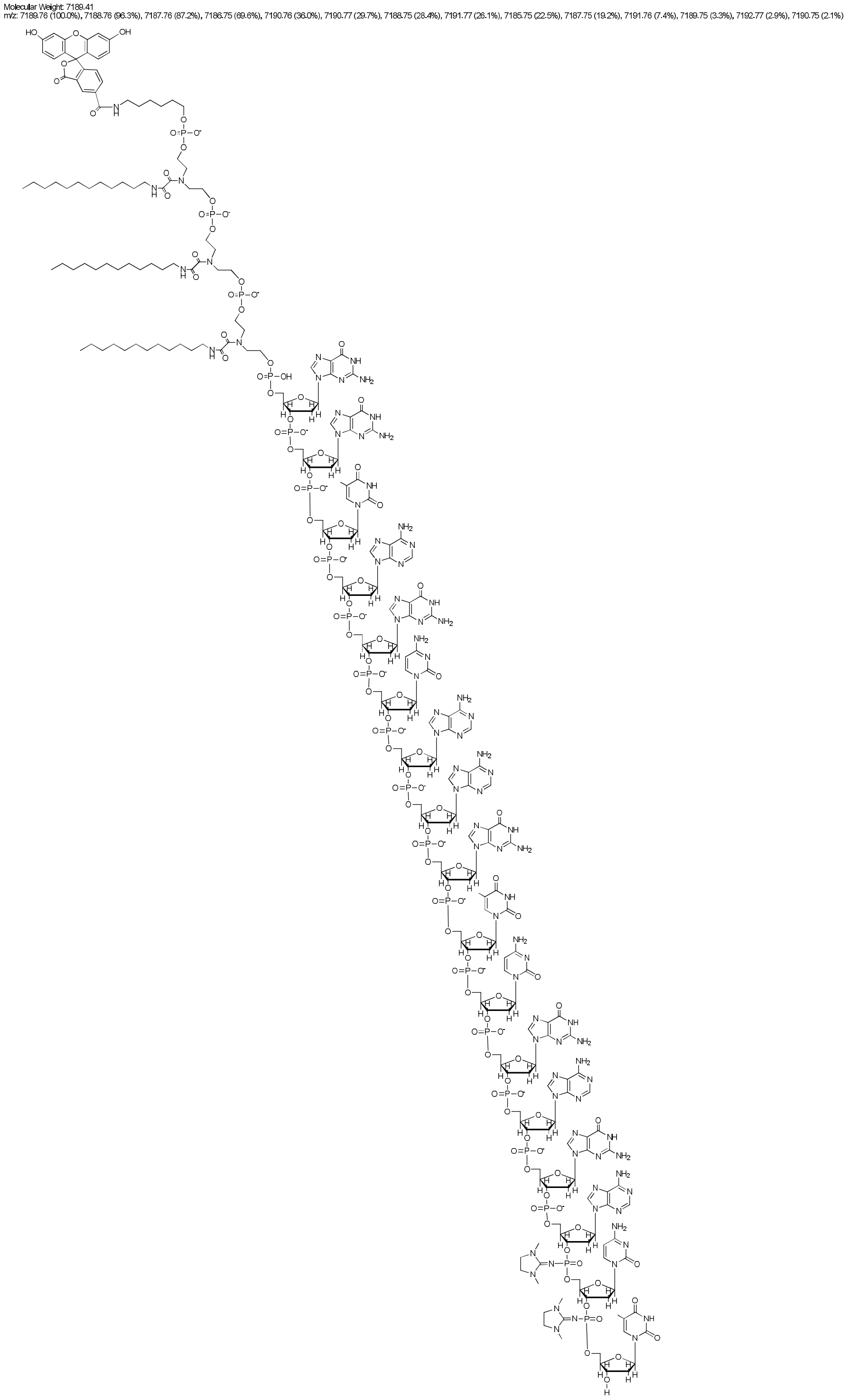

Figure S5. The structure of FAM-D-17^{PG} conjugate.

S1. f. Table S1. Experimental and theoretical molecular masses of the DOCs sythesized

Table S1. Experimental and theoretical molecular masses of the DOCs sythesized.

Oligomer	Calculated value of molecular mass,	Experimental value of molecular mass,

analyzed	g/mole	g/mole
D-13	5549.5	5555.7 (+ Li^{+}) MALDI-TOF
D-13^{PG}	5561.5	5567.8 (+ Li^{+}) MALDI-TOF
D-17	6783.1	6782.0 ESI
D-17^{PG}	6972.5	6978.1 (+ Li^{+}) MALDI-TOF
FAM-D-17	7046.4	7046.5 ESI
FAM-D-17^{PG}	7189.4	7189.7 ESI
FAM-17-D	7315	7323.7 (+ Li^{+}) MALDI-TOF
D-17-FAM	7412	7435.4 (+ Na^{+}) MALDI-TOF
D-22^{PG}	7042.8	7044.1 MALDI-TOF

S1. g. Figure S6. Typical MALDI TOF and ESI mass spectra of DOCs: D-13 (a), FAM-D-17 (b)

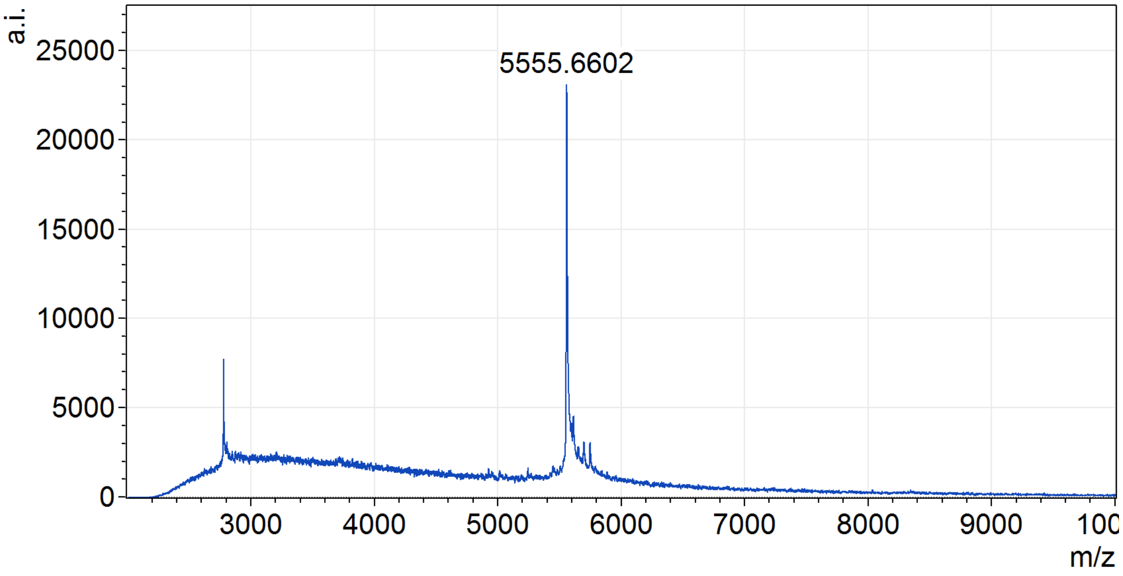

(a)

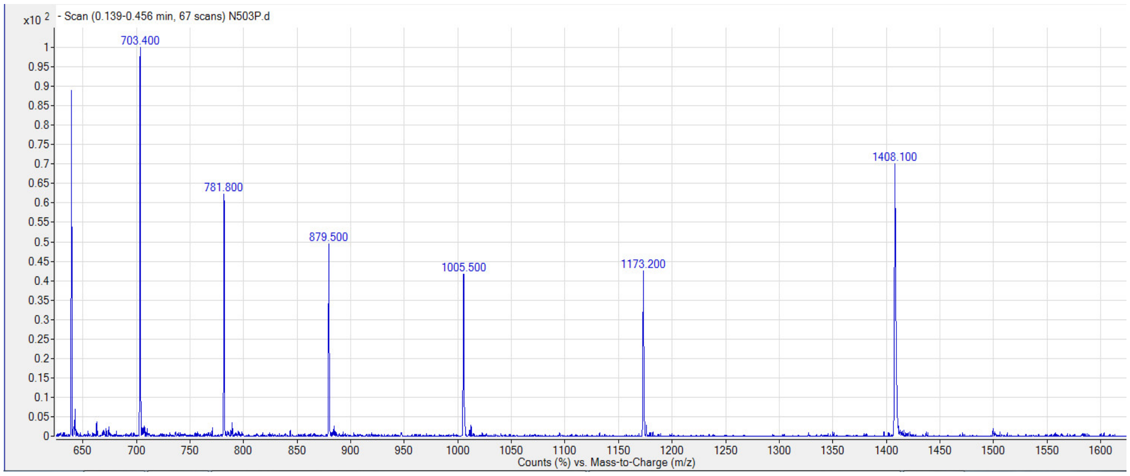

(b)

Figure S6. Typical MALDI TOF and ESI mass spectra of DOCs: D-13 (**a**), FAM-D-17 (**b**).

S2. Self-assembly of 17-mer DOC by Nile Red binding assay

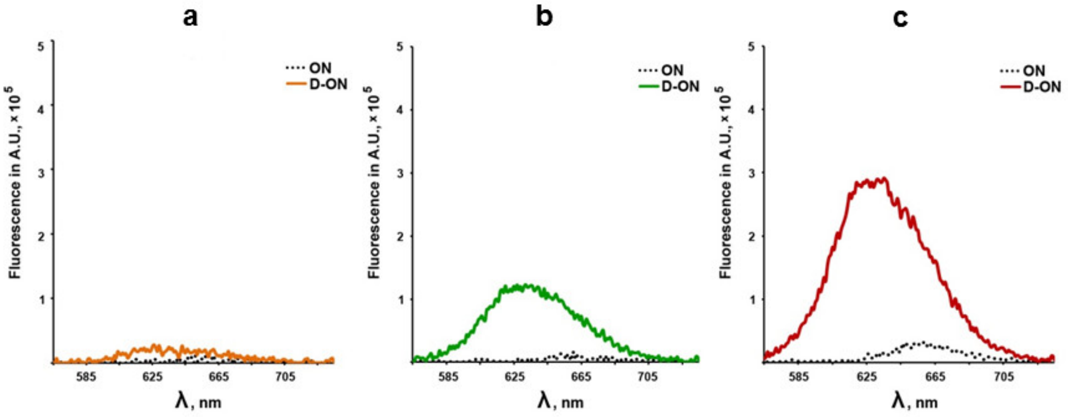

Figure S7. Nile Red fluorescence emission spectra in solutions of indicated concentrations of 17 (ON) and D-17^{PG} (D-ON) in 50 mM Tris-Acetate, pH 8.0, 15 mM $MgCl_2$. Fluorescence intensity of the dye were negliglible without micelles formation near the CAC in solution of DOC (D-17^{PG}, 1 (**a**) μM) and at all concentrations of a control oligonucleotide (17, 1 (**a**), 3 (**b**), and 7 (**c**) μM). Above the CAC value the fluorescence intensity of Nile Red has been rapidly increased D-17^{PG}, 3 (**b**), and 7 (**c**) μM).

S2. Self-assembly of 17-mer DOC by Nile Red binding assay

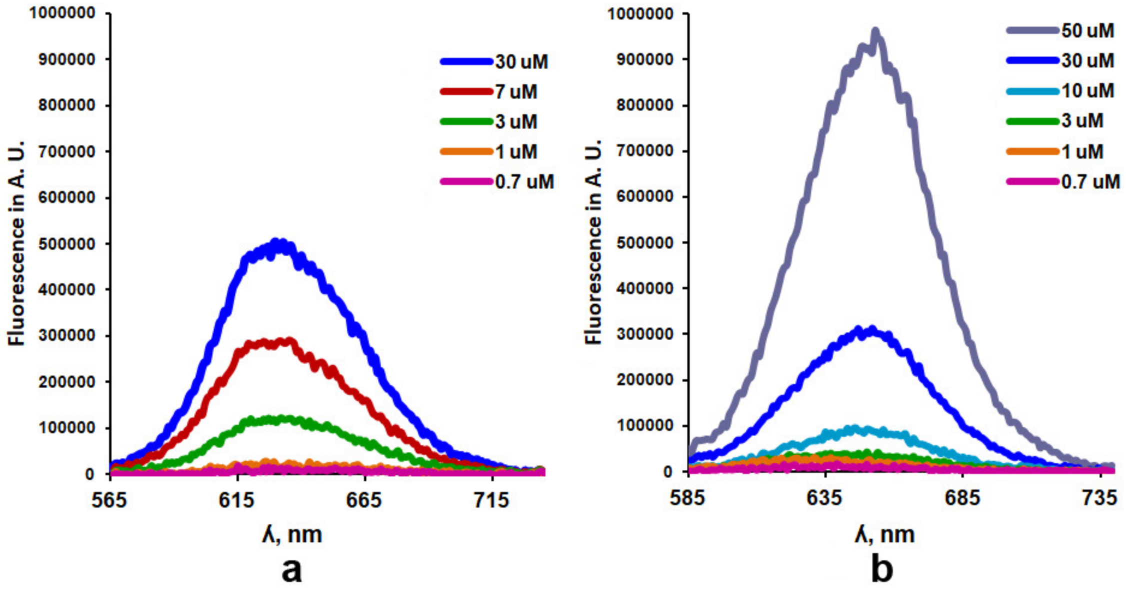

Figure S8. Nile Red fluorescence emission spectra in solutions of varied concentrations D-17^{PG} conjugate in 50 mM Tris-Acetate, pH 8.0, with (**a**) or without (**b**) 15 mM $MgCl_2$.

S3. Dynamic light scattering measurements

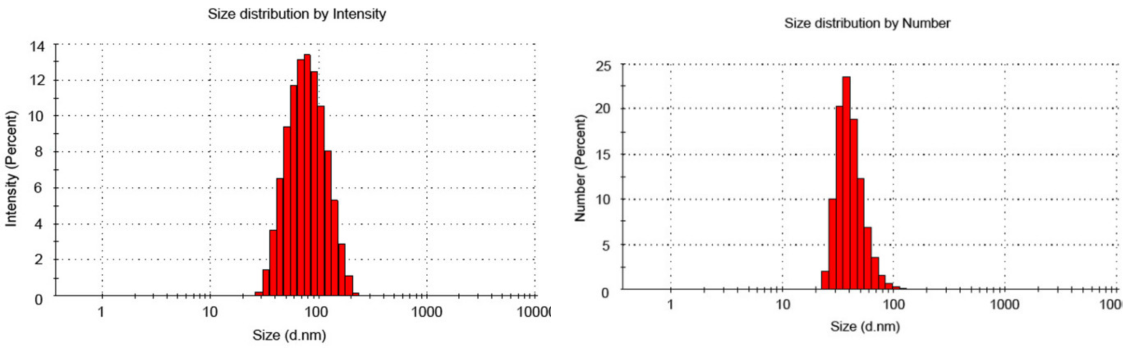

(a) **(b)**

Figure S9. Size distribution by Intensity (**a**) compared with size distribution by Number (**b**) of D-13 conjugate particles after 3 h incubation at 5 μM in 50 mM Tris-Acetate, pH 8.0, 15 mM $MgCl_2$ (TAM buffer) as measured using DLS.

S4. Possible structures of DOCs self-dimers

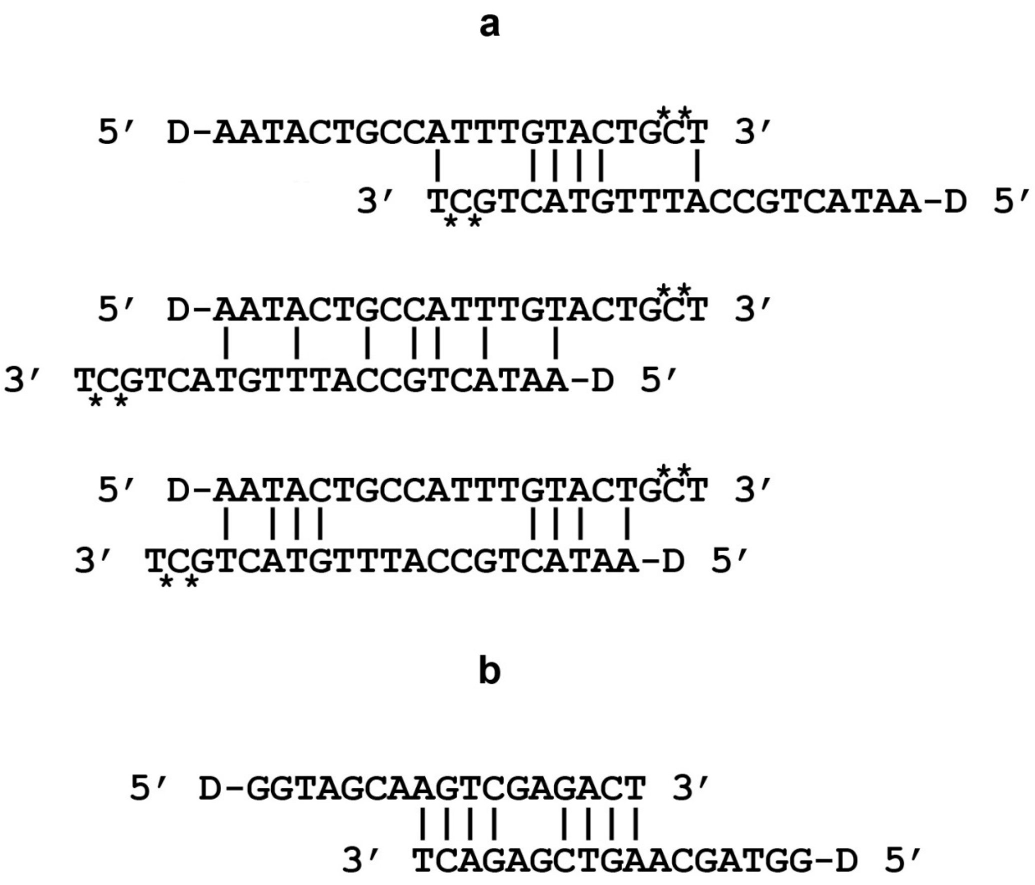

Figure S10. Possible structures of 5′ D-AATACTGCCATTTGTACTG*C*T 3′ (**a**) and D-17 (**b**) partial self-dimers.

S5. AFM additional images

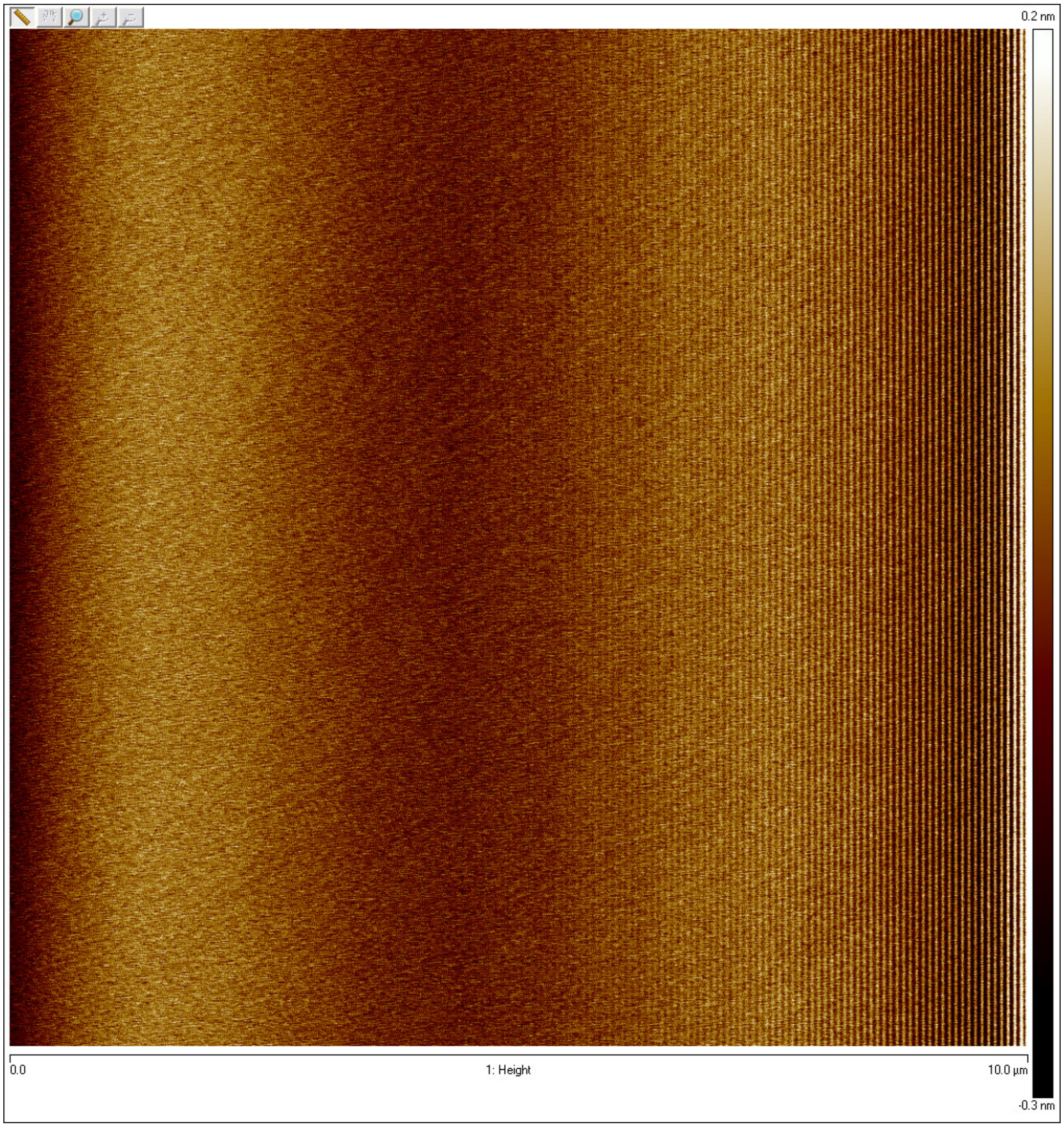

Figure S11. Mica surface.

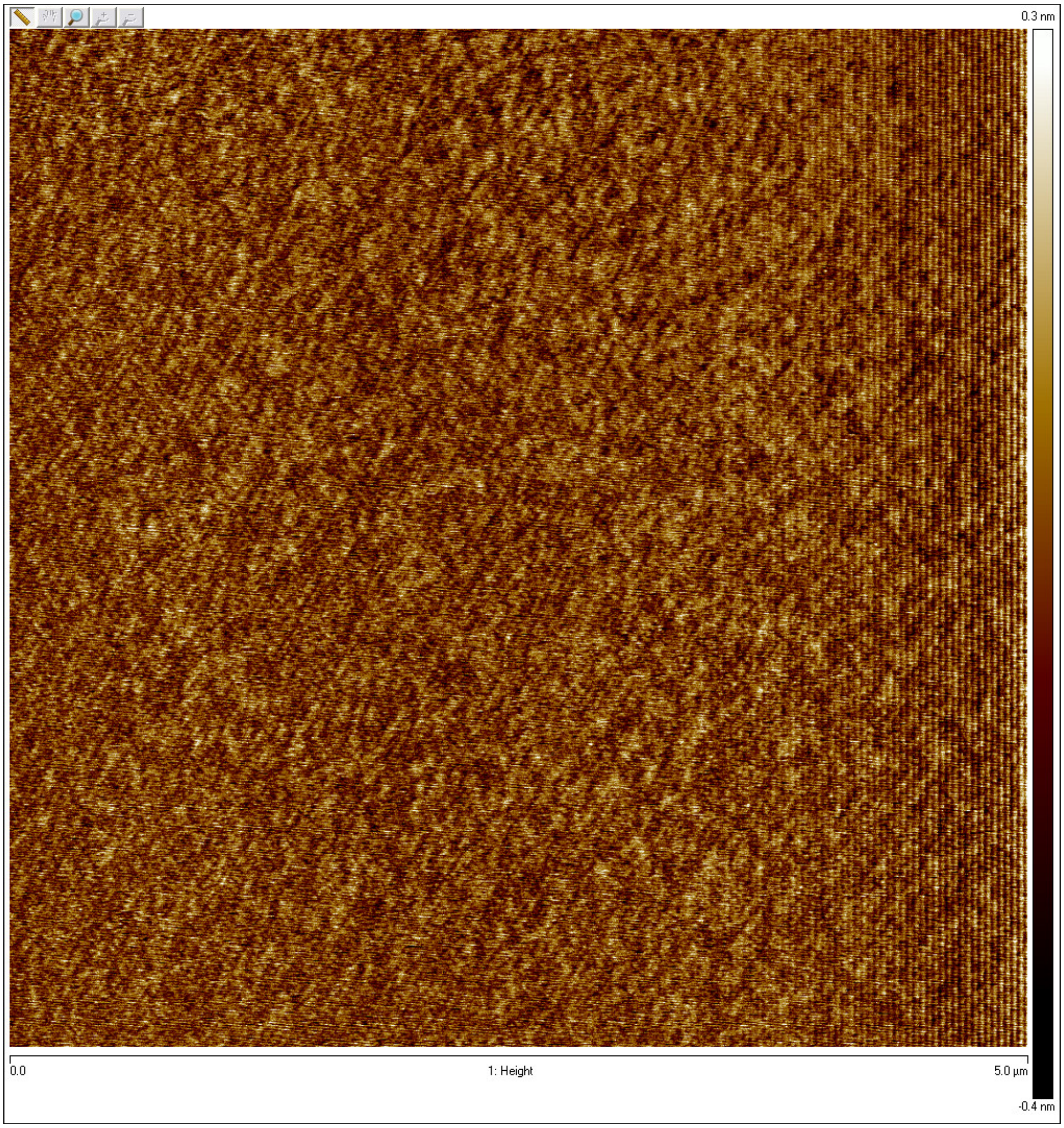

Figure S12. AFM control image of water deposited onto a freshly cleaved mica.

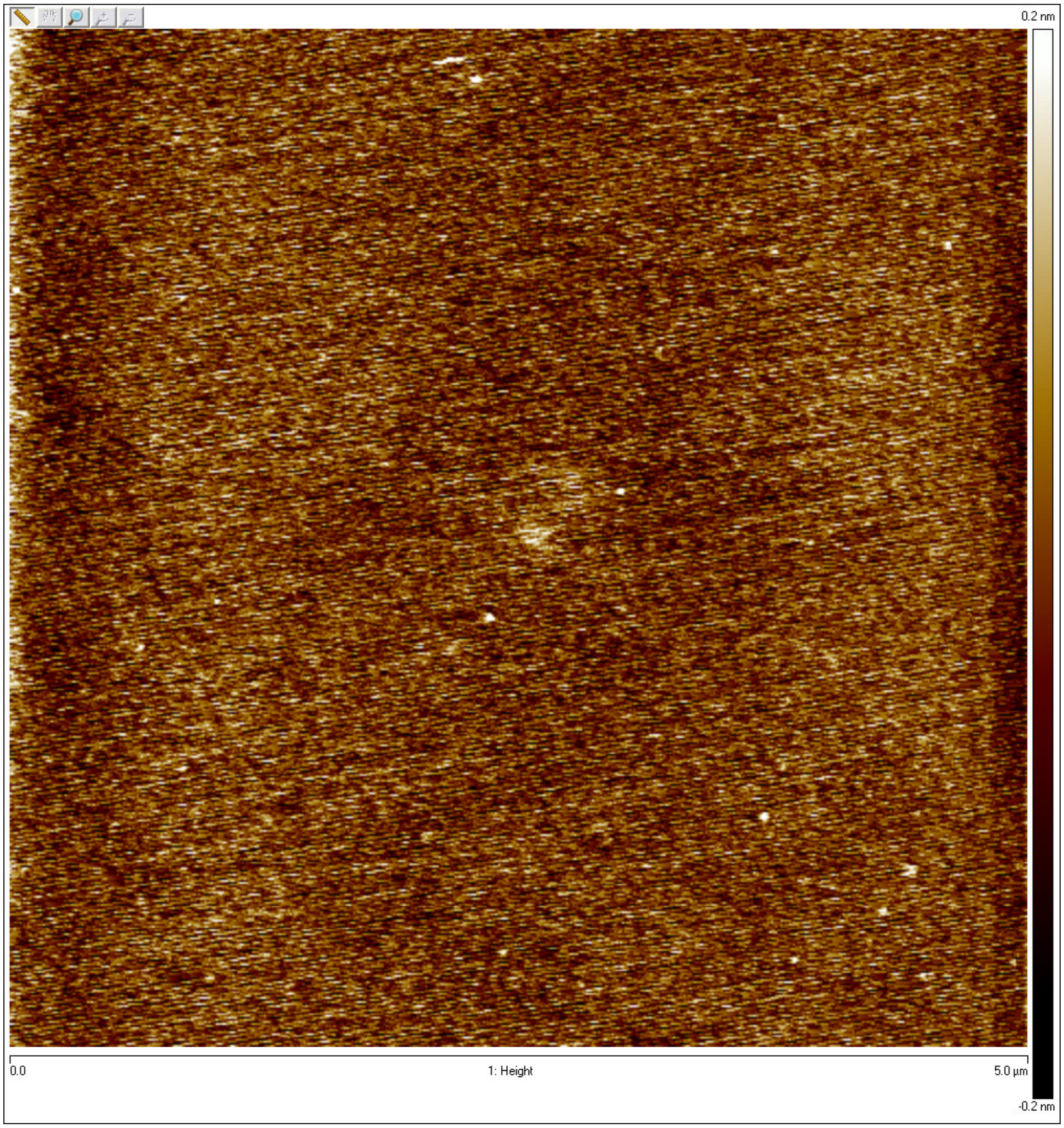

Figure S13. AFM control image of 17 oligonucleotide without dodecyl chains in water.

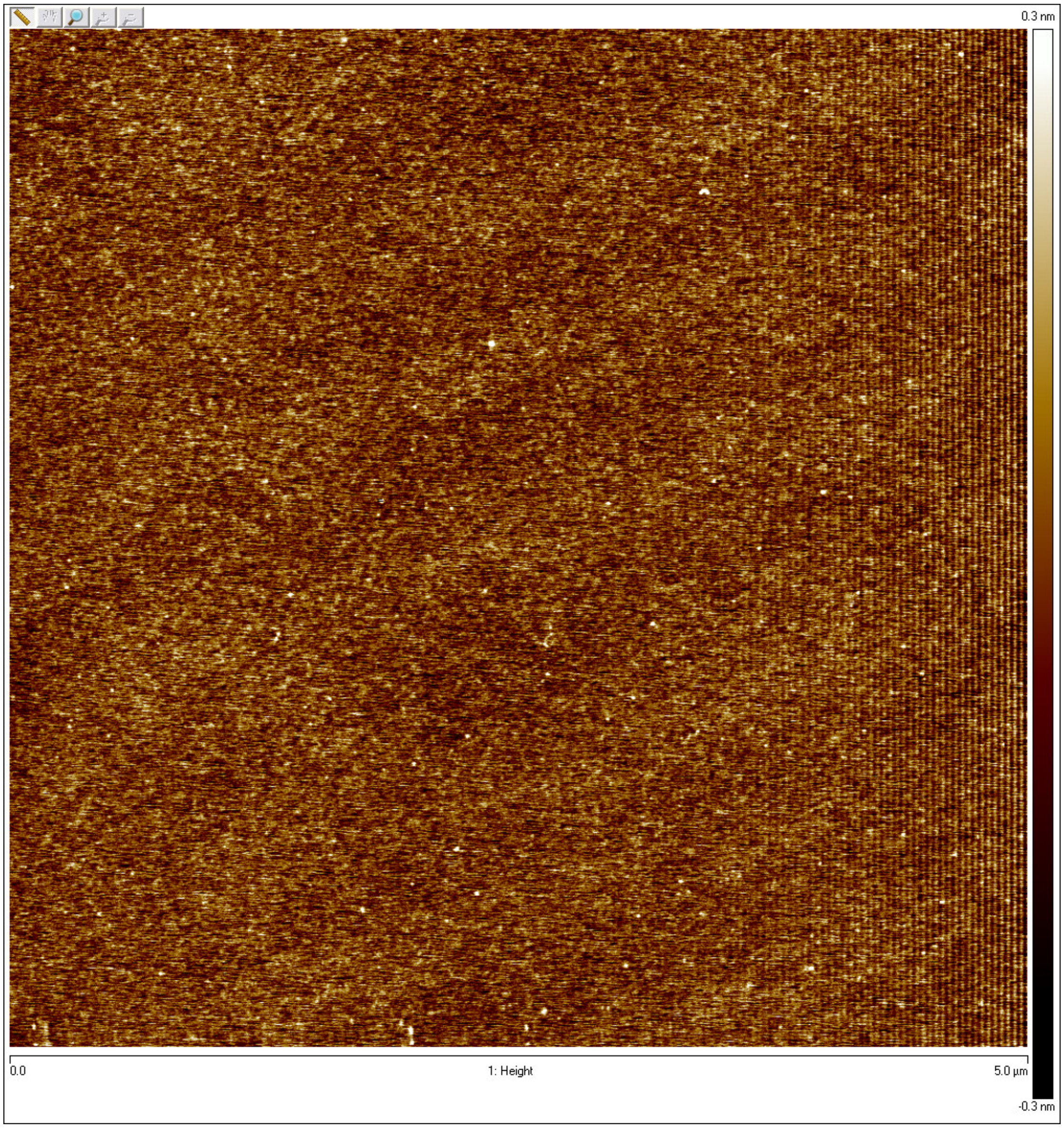

Figure S14. AFM control image of TAM buffer.

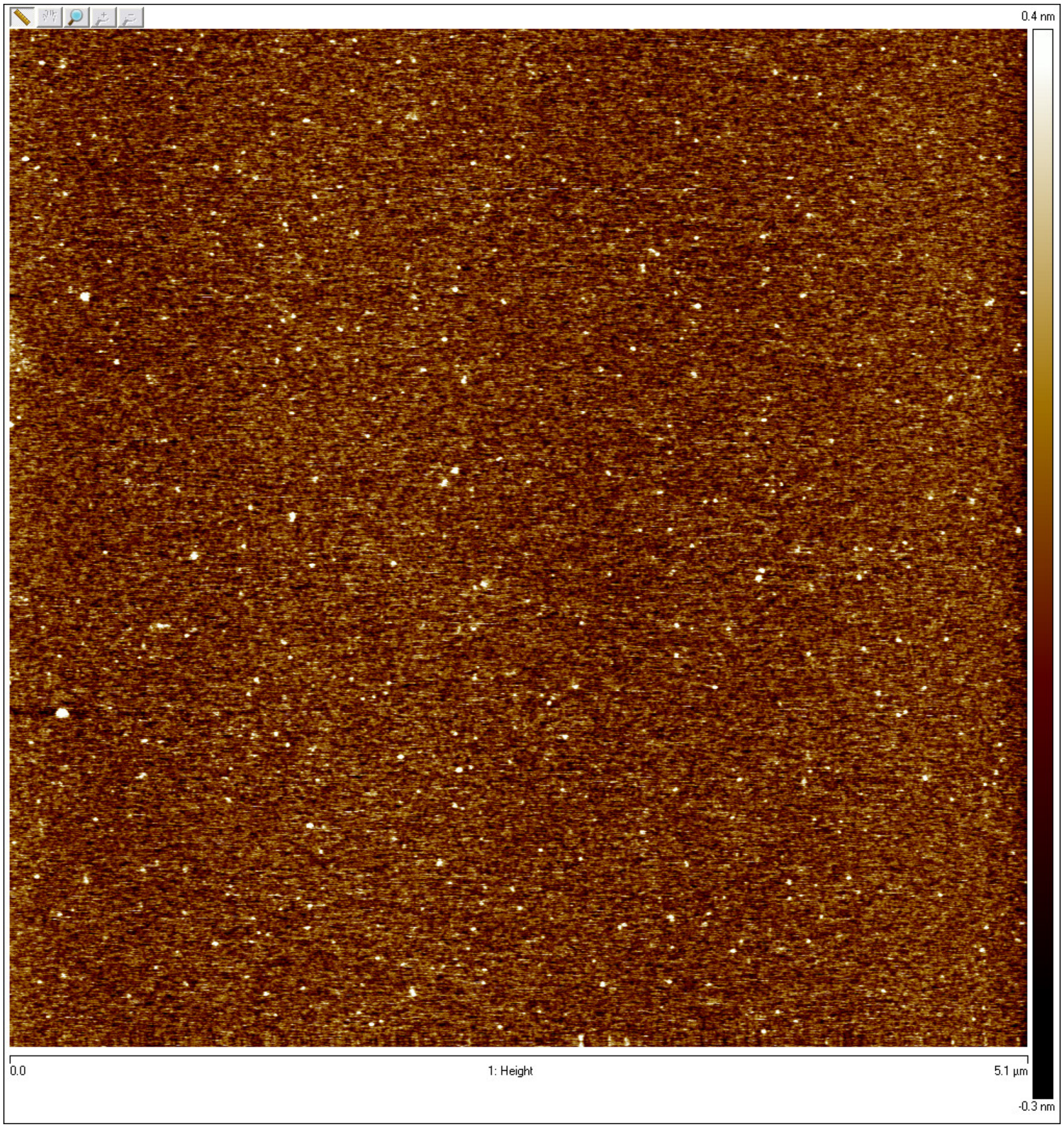

Figure S15. AFM image of 17:17′ duplex in TAM.

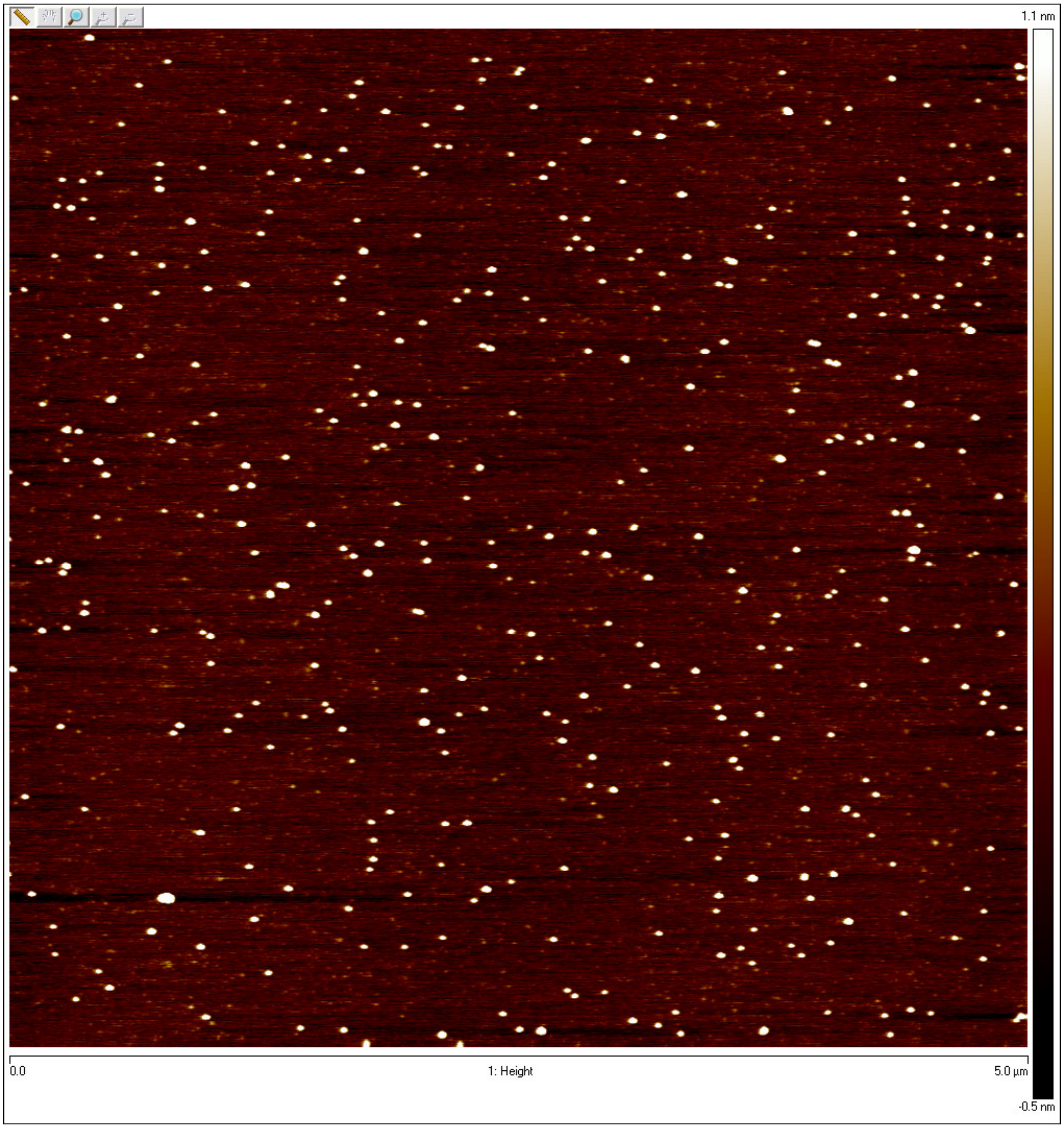

Figure S16. AFM image of D-17 in TAM.

S6. TEM additional images

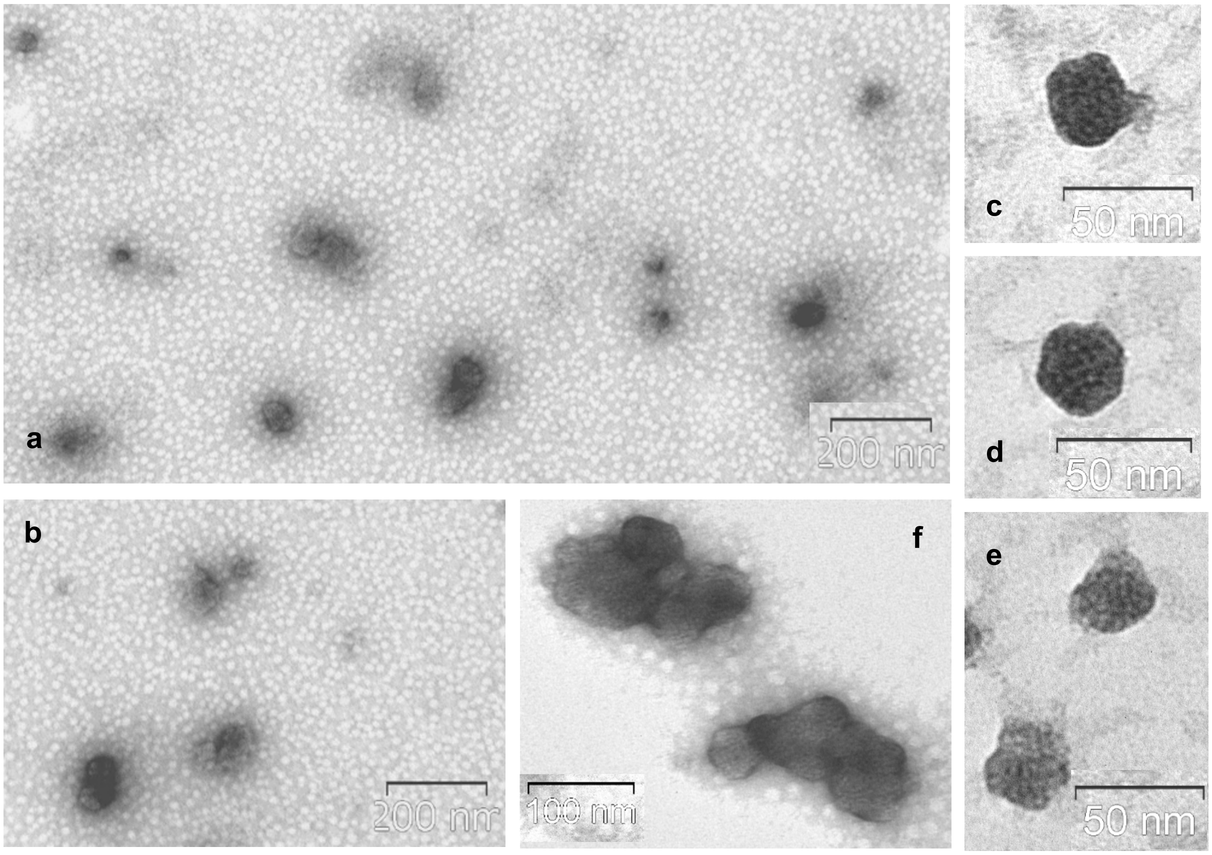

Figure S17. Representative TEM images of D-17 micellar particles in TAM buffer. (**a**–**b**)—common view; (**c**–**e**)—individual particles; (**f**)—aggregates of the particles. Negative staining with uranyl acetate. Scale bar is indicated.

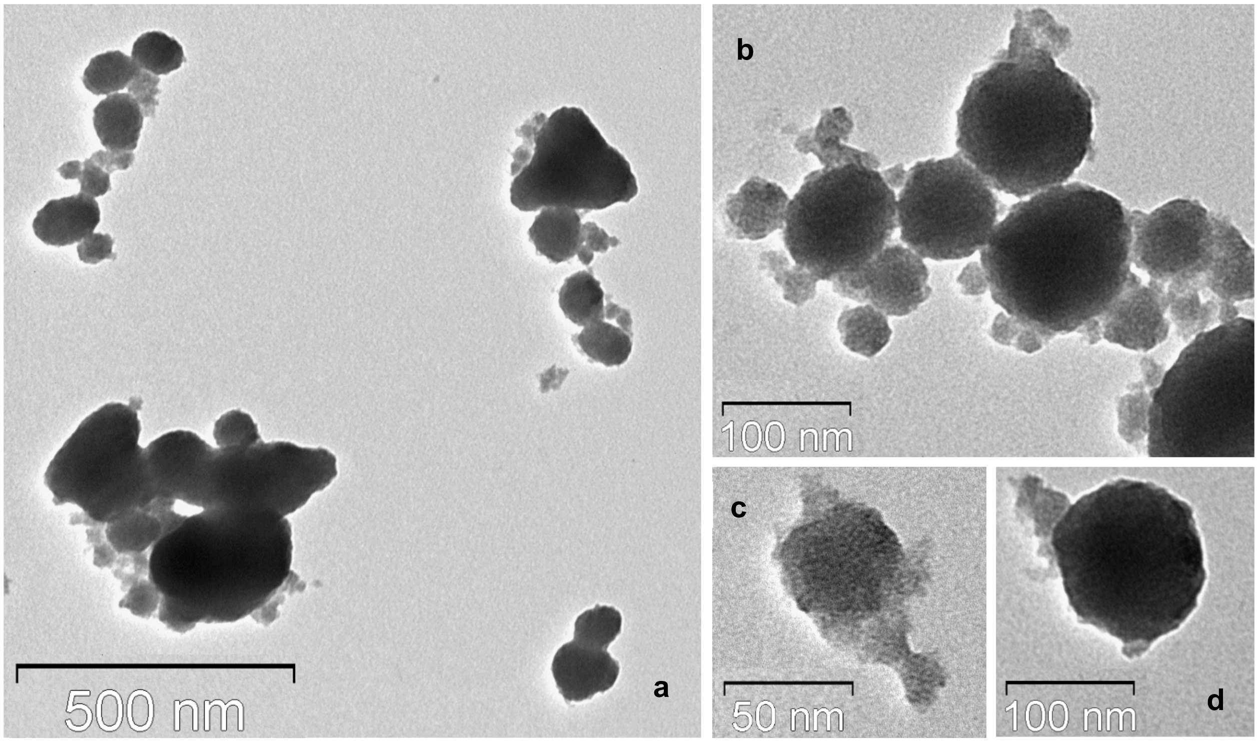

Figure S18. Representative TEM images of D-17^{PG} micellar particles in TAM buffer. (**a**–**b**)—aggregates of the particles; (**c**–**d**)—individual particles. Negative staining with uranyl acetate. Scale bar is indicated.

S7. BSA binding with D-13^{PG} conjugate

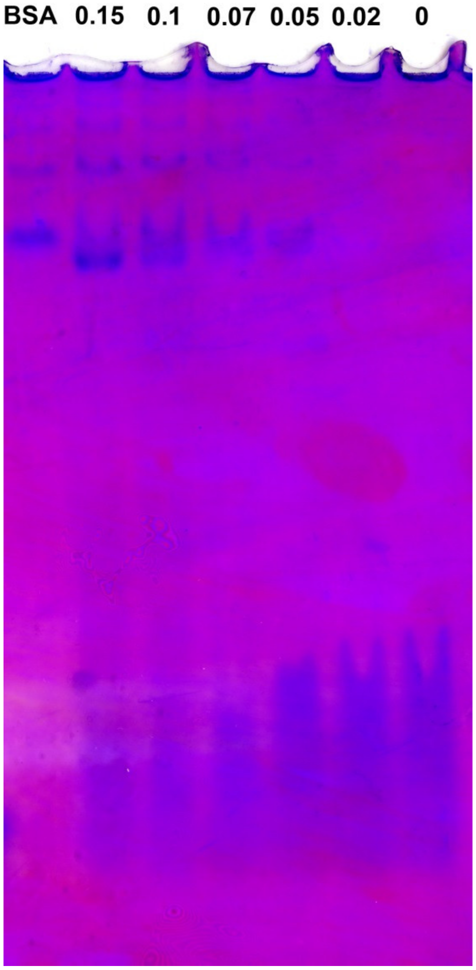

Figure S19. Comparative electrophoretic mobility of a bovine serum albumin as a control (BSA lane, 3.75 µM of the protein without any oligonucleotides in the probe) and with D-13^{PG} conjugate (0.15–0.02 corresponds to the excess of the protein relative to the oligomer) in the probes. The reaction mixtures were investigated by non-denaturing 8% PAGE after 3 h incubation at 37 °C in TAN buffer. Each sample in 0.15–0.02 lanes contained 25 µM of the corresponding oligomer. Bands were visualized by Stains-All staining.

S8. **Fluorescence quenching experiments**.

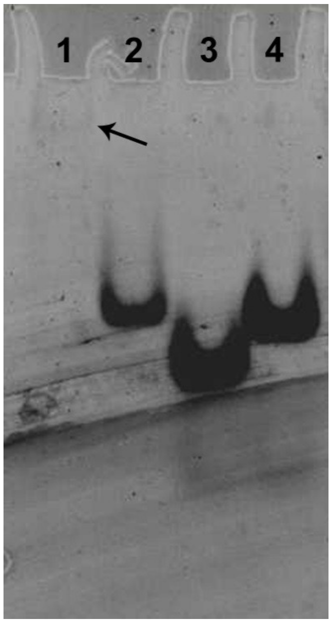

Figure S20. Comparative electrophoretic mobility of the FAM-D-17 DOC (Lane 1), FAM-D-17/17′ duplex (Lane 2), FAM-17′ (Lane 3), and FAM-17′/17 duplex (Lane 4) investigated by non-denaturing 10% PAGE after 3 h incubation in 0.89 M Tris-Borate, pH 8.3, with 5 mM $MgCl_2$, at 25 °C; each sample contained 2 μM of all corresponding oligomers. Representative gel image was recorded after scanning using Pharos FX Molecular Imager System (Bio-Rad, Hercules, CA, USA) (scanning parameters: laser excitation wavelength 488 nm and emission filter 530 nm).

S9. Absorbance spectra of FAM-D-17, FAM-17-D, D-17-FAM

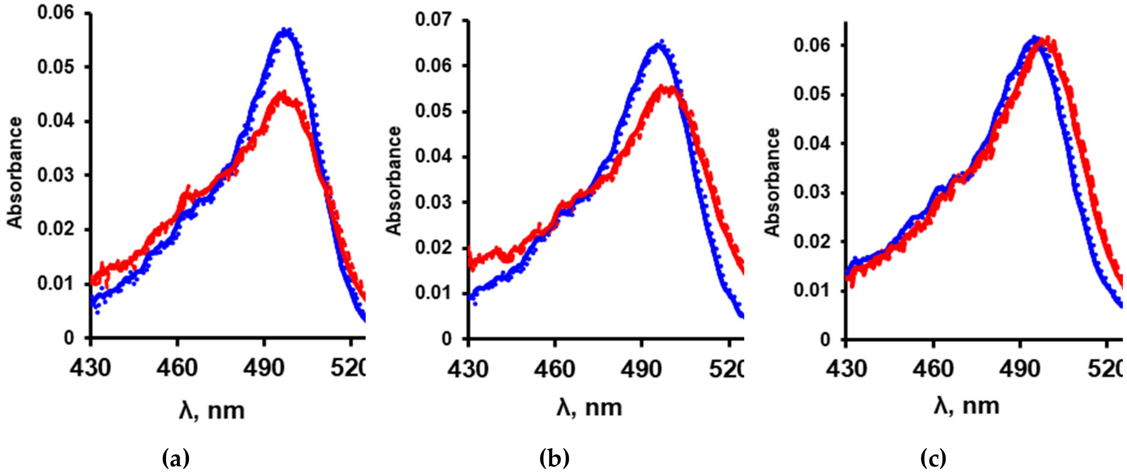

Figure S21. Absorbance spectra (dashed lines) with added trend lines (solid lines) of 1μM FAM-D-17 (**a**), FAM-17-D (**b**), D-17-FAM (**c**), DOCs in aqueous solution either without (blue lines) or with (red lines) 15 mM $MgCl_2$.

S10. The stability of FAM-D-17^{PG} conjugate and FAM-17′/D-17^{PG} duplex used for transfection in 10% FBS

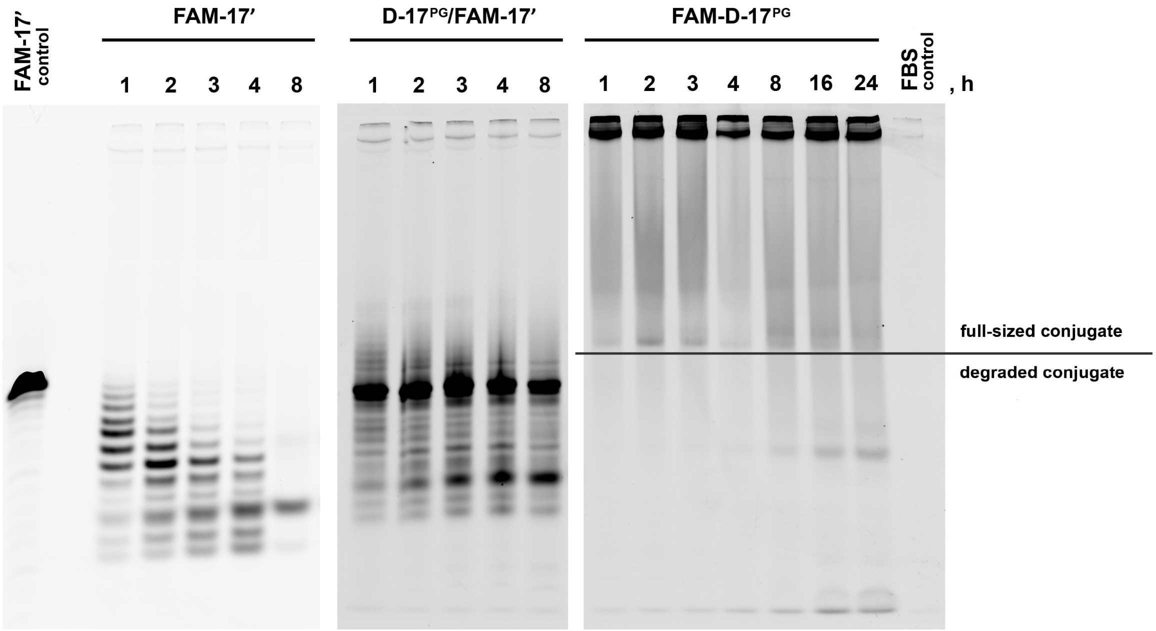

Figure S22. The analysis of products of control oligonucleotide (FAM-17′), DOC (FAM-D-17^{PG}) and duplex (FAM-17′/D-17^{PG}) degradation in 10% FBS by denaturing PAGE (15% PAAG, 8 M urea, 0.089 M Tris, 0.089 M boric acid, pH 8.3). 5 μM of each sample was treated with 10% FBS in DMEM. After incubation at indicated points of time and 37 °C the probes were precipitated, diluted in 8M urea with XC, and analyzed by PAGE. The lane of FAM-17′ control represented a pattern of FAM-17′ oligonucleotide band in DMEM after 24 h incubation. The lane of FBS control represented a pattern of 10% FBS in DMEM without nucleic acids.

As can be seen from the FigureS22, fluorescently labeled oligodeoxynucleotide FAM-17′ completely degraded after 8 h in 10% FBS. Both of the transfected samples (a dodecyl oligonucleotide conjugate FAM-D-17^{PG} and the duplex FAM-17′/D-17^{PG}) are more stable. After 8 h incubation of FAM-17′/D-17^{PG} in 10% FBS the extent of its degradation was about of 50% (Figure S22). Interestingly, FAM-D-17^{PG} conjugate has such a great affinity for serum proteins binding that even in denaturing conditions it is in wells in the gel in a complex with proteins. It is difficult to calculate accurately the extent of the conjugate degradation in this case, but FAM-D-17^{PG} degraded bands do not exceed 12.5% of the total fluorescence signal in the lane even after 24 h of incubation (Figure S22).
